# Network-guided search for genetic heterogeneity between gene pairs

**DOI:** 10.1093/bioinformatics/btaa581

**Published:** 2020-06-23

**Authors:** Anja C Gumpinger, Bastian Rieck, Dominik G Grimm, International Headache Genetics Consortium, Karsten Borgwardt

**Affiliations:** Department of Biosystems Science and Engineering, ETH Zürich, Basel 4058, Switzerland; SIB Swiss Institute of Bioinformatics, Lausanne 1015, Switzerland; Department of Biosystems Science and Engineering, ETH Zürich, Basel 4058, Switzerland; SIB Swiss Institute of Bioinformatics, Lausanne 1015, Switzerland; Technical University of Munich, TUM Campus Straubing for Biotechnology and Sustainability, Bioinformatics, Straubing 94315, Germany; Weihenstephan-Triesdorf University of Applied Sciences, Bioinformatics, Straubing 94315, Germany; Department of Biosystems Science and Engineering, ETH Zürich, Basel 4058, Switzerland; SIB Swiss Institute of Bioinformatics, Lausanne 1015, Switzerland

## Abstract

**Motivation:**

Correlating genetic loci with a disease phenotype is a common approach to improve our understanding of the genetics underlying complex diseases. Standard analyses mostly ignore two aspects, namely genetic heterogeneity and interactions between loci. Genetic heterogeneity, the phenomenon that genetic variants at different loci lead to the same phenotype, promises to increase statistical power by aggregating low-signal variants. Incorporating interactions between loci results in a computational and statistical bottleneck due to the vast amount of candidate interactions.

**Results:**

We propose a novel method SiNIMin that addresses these two aspects by finding pairs of interacting genes that are, upon combination, associated with a phenotype of interest under a model of genetic heterogeneity. We guide the interaction search using biological prior knowledge in the form of protein–protein interaction networks. Our method controls type I error and outperforms state-of-the-art methods with respect to statistical power. Additionally, we find novel associations for multiple *Arabidopsis thaliana* phenotypes, and, with an adapted variant of SiNIMin, for a study of rare variants in migraine patients.

**Availability and implementation:**

Code available at https://github.com/BorgwardtLab/SiNIMin.

**Supplementary information:**

Supplementary data are available at *Bioinformatics* online.

## 1 Introduction

Testing associations between individual genetic markers and a disease phenotype has shown large successes for Mendelian diseases, where individual point mutations at one genetic locus cause an individual to develop a disease phenotype (e.g. Kerem *et al.*, 1989; MacDonald *et al.*, 1992). However, for complex diseases that presumably result from a complex interplay between genetic markers, environmental factors and lifestyle (Hunter, 2005), genetic loci detected in univariate association studies often fail to explain the phenotypic variation. This phenomenon is referred to as *missing heritability* (Lee *et al.*, 2011; Manolio *et al.*, 2009; Visscher *et al.*, 2008, 2012). Focusing on interactions between genetic markers promises to increase our understanding of disease mechanisms and address the problem of missing heritability (Zuk *et al.*, 2012). One such mode of interaction is *genetic heterogeneity*, meaning that different genetic variants affect a phenotype in a similar direction (McClellan and King, 2010). While technical advances allow genotyping individuals at ever-increasing resolution, this complicates the search for joint effects between individual genetic variants, as the number of potential interactions explodes with the number of genetic variants. We refer to the genetic variants as *features* in the following. A common approach is pairwise testing of single nucleotide polymorphisms (SNPs), for example, to test for epistatic effects (Cordell, 2002). This pairwise search scales quadratically with the number of features, posing not only a computational but also a statistical challenge in the form of a multiple hypothesis testing burden. If not accounted for, the multiplicity of tests can lead to high numbers of associations with the phenotype by random chance. Especially if they are to be evaluated in follow-up studies, strictly avoiding large numbers of false positives is indispensable. This is achieved by controlling the familywise error rate (FWER), i.e. the probability to observe false positives. While the classical Bonferroni correction (Bonferroni, 1936) accomplishes this, it is too conservative and avoids false positives at the expense of accepting large numbers of false negatives. Tarone (1990) showed that a less restrictive control of the FWER exists that increases statistical power for discrete tests. This observation has been used extensively in *significant pattern mining* to simultaneously alleviate the statistical and computational challenge imposed by testing large amounts of hypotheses (Llinares-López *et al.*, 2015a; Minato *et al.*, 2014; Papaxanthos *et al.*, 2016; Terada *et al.*, 2013). Recently, Tarone’s procedure has shown success in various bioinformatics disciplines (Bock *et al.*, 2018; Llinares-López *et al.*, 2015b, 2017, 2019). Despite those advances, mining pairwise SNP interactions in datasets with high-dimensional feature spaces in a statistically rigorous manner remains an open challenge. A different line of research aims at elucidating genetic interactions by adopting a gene-based view (Kwon *et al.*, 2018; Povysil *et al.*, 2019; Zhao *et al.*, 2016), where genes are represented as variant-sets. While this is certainly promising, those methods might be underpowered if not the interaction between whole genes, but only sub-regions within the genes, are driving the association. At the same time, large numbers of high-quality biological networks describing various relationships between genes (or their products) have become available for different organisms (e.g. Li *et al.*, 2017; Luijk *et al.*, 2018; Kanehisa *et al.*, 2017; Obayashi and Kinoshita, 2011). Including feature relationships encoded in these networks has boosted biological analyses in different areas (e.g. Azencott *et al.*, 2013; Horn *et al.*, 2018; Krogan *et al.*, 2015; Shen *et al.*, 2017; Zhang *et al.*, 2018). Hence, having seen the advantages of biological networks, combining them with concepts from significant pattern mining and Tarone’s procedure to guide the search for interactions seems promising. This article proposes *Significant Network-Interaction Mining* (SiNIMin), a method that combines Tarone’s procedure with concepts from significant pattern mining and biological networks to detect pairs of genes that exhibit association with a phenotype. SiNIMin integrates biological networks with SNP data to test whether pairwise interactions between gene segments are associated with a phenotype of interest under a model of genetic heterogeneity. Since testing all such pairwise interactions is computationally and statistically intractable, we leverage networks to restrict the search space to combinations of segments coming from two different genes that share an edge. Combining this interaction-mining approach with techniques from significant pattern mining makes solving a previously intractable problem feasible. Our approach has multiple advantages: (i) The statistical and technical tricks from the pattern mining field alleviate the intrinsic multiple-hypothesis testing problem and provide an efficient criterion to prune the search space of interactions. (ii) The combination of segments within interacting genes generates biologically meaningful hypotheses. (iii) By analyzing the sub-parts of genes, as opposed to whole genes, edges or modules in a network, we get a ‘close-up view’ of the genetics underlying disease at the highest resolution, namely at the level of individual bases. (iv) With the assumption that various SNPs might influence a phenotype in a similar way, we gain additional power by aggregating them under a model of genetic heterogeneity (Llinares-López *et al.*, 2015b, 2017).

## 2 Materials and methods

### 2.1 Problem statement

**Fig. 1.**
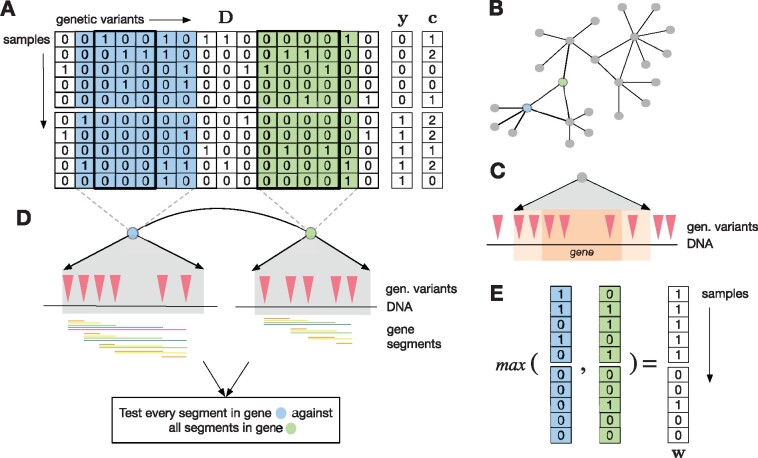
(**A**) The binary dataset **D**. Rows correspond to samples, columns correspond to binary genetic variants. Phenotype vector **y** and categorical covariate vector **c**. (**B**) Network. Nodes correspond to genes, and each edge represents an interaction between two genes. (**C**) Mapping of genetic variants to genes in the network. All variants that overlap with a gene’s introns and exons (dark orange) are mapped to the gene. Optionally, variants that lie in a fixed-size window around the gene (light orange) are mapped to the gene as well. (**D**) Analysis of an interaction. Both adjacent genes are represented by their overlapping genetic markers, highlighted in blue and green in the data matrix. All possible segments are considered in both genes (illustrated by horizontal colored bars, where segments of length 1, i.e. single SNPs, are omitted). Each segment from the first gene is tested against each segment from the second gene. (**E**) Heterogeneity encoding (allelic heterogeneity) of two segments in the blue and green genes, highlighted with black boxes in A. In white, heterogeneity encoding (locus heterogeneity) **w** of their interaction. The *max* function is the elementwise maximum over the two vectors

Consider a study of *n* samples, each coming from one of two phenotypic classes. We store this class assignment in an *n*-dimensional, binary phenotype vector $y\in {\left\{0,1\right\}}^{n}$. Furthermore, each of the *n* samples is represented by a *d*-dimensional binary genotype that could correspond to genetic variants, such as SNPs or rare variants in a dominant encoding. The genotypes are stored in a data matrix $D\in {\left\{0,1\right\}}^{n\times d}$. Optionally, there may be a discrete covariate vector $c\in {\left\{1,\ldots ,k\right\}}^{n}$ that assigns each sample to one of *k* discrete covariate classes (see Fig. 1A). In addition, we have information about interactions between genetic regions in the form of a network. We represent this network formally as G, consisting of a node set V and an edge set E, that is G=(V,E). An example are protein–protein interaction (PPI) networks, where nodes correspond to gene products, and edges correspond to interactions between them (see Fig. 1B). Each gene can be represented by the set of variants that overlap with its exons and introns. Optionally, variants that fall into a window up- and downstream of the gene can be included in the representation (see Fig. 1C). We define a genetic segment as a range of subsequent variants along the sequenced genome and denote it with the tuple (*s*, *l*), where *s* corresponds to the starting position of the segment, that is its first variant, and *l* is its length. We restrict ourselves to segments within genes, and call them *gene segments* in the following.

Our goal is to find pairs of gene segments that (i) show a statistically significant association with the binary phenotype **y** under a model of genetic heterogeneity and that (ii) can be mapped to an edge in the network, that is, each segment comes from one of two genes, with genes connected by an edge in G. This poses three challenges: (i) enumeration of all possible segments of genetic variants within any two genes that share an edge, (ii) testing of all possible combinations of segments from both genes (see Fig. 1D) and (iii) correcting for the dependency structure between gene segment interactions, arising due to the extensive overlap between the segments. These challenges can be addressed using concepts from the field of *significant pattern mining*.

### 2.2 Patterns as interactions between gene segments

A pattern is generally defined as a discrete subset of the *d* features in the data matrix **D**, ranging from single features to large combinations. Here, we restrict ourselves to interactions between gene segments, and we will use the terms *segment interaction* and *pattern* interchangeably. Importantly, patterns exhibit sub- and super-structure relationships. For example, the interaction between segments (*s*_1_, *l*_1_) and (*s*_2_, *l*_2_) is a *sub-pattern* of the interaction between $\left(s_{1},l_{1}+1\right)$ and (*s*_2_, *l*_2_), as it is fully contained in the latter. Inversely, the interaction between segments $\left(s_{1},l_{1}+1\right)$ and (*s*_2_, *l*_2_) is a *super-pattern* of the interaction between segments (*s*_1_, *l*_1_) and (*s*_2_, *l*_2_). Similarly, the interaction between segments (*s*_1_, *l*_1_) and (*s*_2_, *l*_2_) can be expanded in any other direction, giving rise to a multitude of super-patterns.

To test the combination of two gene segments (*s*_1_, *l*_1_) and (*s*_2_, *l*_2_) from interacting genes *g*_1_ and *g*_2_, respectively, we first represent each segment as a sub-matrix of the data matrix of dimension $n\times |l_{i}|,i\in \{1,2\}$ (see black boxes in Fig. 1A). We reduce each segment to an *n*-dimensional vector $m^{\left(s,l\right)}\in {\left\{0,1\right\}}^{n}$ where $m_{j}^{\left(s,l\right)}=\max(D_{j,s:s+l})$ and j=1,…,n indexes the samples. If for sample *j* any variant in the segment has value 1, its representation will also be 1, and we call this *heterogeneity encoding* (see also Li and Leal, 2008; Morris and Zeggini, 2010). The combination represents allelic heterogeneity within the gene since multiple variants in a segment lead to the same effect on the final representation (Fig. 1E). To combine two segments $\left(s_{1},l_{1}\right)$ and (*s*_2_, *l*_2_), we merge their representations $m^{\left(s_{1},l_{1}\right)}$ and $m^{\left(s_{2},l_{2}\right)}$, again using the above-mentioned heterogeneity encoding. This combination gives rise to an *n*-dimensional vector $w^{\left(s_{1},l_{1}),(s_{2},l_{2}\right)}\in {\left\{0,1\right\}}^{n}$, representing locus heterogeneity. For simplicity of notation, we drop the indices because they will be clear from the context, and denote the encoding as **w** throughout this section. While other popular combinations of variants, such as burden tests, exist, the heterogeneity encoding introduced here is a biologically interesting concept (McClellan and King, 2010), that also constitutes a key-component of our algorithmic contribution: given the binary representation of a segment interaction and the phenotype, contingency-based tests, such as Fisher’s exact test, Pearson’s $\chi^{2}$ test (Pearson, 1900) or a Cochran-Mantel–Haenszel test (CMH, Mantel and Haenszel, 1959) can be used to measure association. Importantly, to reduce the redundancy between patterns, we enumerate segments in a closed form: if a segment has the same heterogeneity encoding as any of its sub-patterns, it will not be enumerated.

### 2.3 Statistical testing and control of the FWER

Up to now, we have established how to define, represent and combine gene segments. The binary reformulation of the problem by means of the heterogeneity encoding allows us to exploit several concepts from the field of significant pattern mining (Minato *et al.*, 2014; Papaxanthos *et al.*, 2016; Terada *et al.*, 2013). Those methods rely on the concept of the *minimum p-value*.

#### 2.3.1 The minimum *p*-value

Given a pattern’s binary encoding **w** and the phenotype vector **y**, we can generate a contingency table such as in Table 1 as the basis for a discrete test. For fixed table marginals *x, n* and *n*_1_, the table has exactly one degree of freedom (w.l.o.g., we use the table count *a*). Since every value of *a* leads to a contingency table with a specific *p*-value, there exists a value of *a* that leads to the *minimum p-value* of the table, and this minimum *p*-value can be computed analytically as a function of the table margin *x* (Llinares-López *et al.*, 2015a; Papaxanthos *et al.*, 2016). Here, we focus on the CMH test, which can account for categorical covariates by creating one separate contingency table for every covariate class *k* [see Papaxanthos *et al.* (2016) for details]. However, all approaches can easily be extended to Fisher’s exact test and the $\chi^{2}$ test.

**Table 1.** A 2 × 2 contingency table to test the binary encoding **w** of a segment interaction $\left(s_{1},l_{1}),(s_{2},l_{2}\right)$ for its association with the binary phenotype **y**

Class label	*w *=* *1	*w *=* *0	Row totals
*y* = 1	*a*	$n_{1}-a$	*n*_1_
*y* = 0	*x* – *a*	$n-n_{1}-x+a$	$n-n_{1}$
Col. totals	*x*	*n* – *x*	*n*

#### 2.3.2 Tarone’s procedure to control the FWER

Tarone’s procedure (Tarone, 1990) is a method to control the FWER, that is to restrict the probability of observing one or more false-positive associations when large numbers of tests are conducted simultaneously. The FWER is controlled at a significance level *α*, i.e. one finds a per-hypothesis threshold *δ*, such that FWER(δ)≤α. To maximize statistical power, one determines an optimal $\delta^{*}=\text{max}\{\delta \;|\;\text{FWER}(\delta )\leq \alpha \}$. A common approach for FWER-control is the Bonferroni correction (Bonferroni, 1936), where the target significance level *α* is divided by the number of simultaneous tests, resulting in a per-test significance threshold of $\delta_{\text{bon}}=\alpha /m$, where *m* is the number of tests. For a large number of tests, we have $\delta_{\text{bon}}\ll \delta^{*}$; the Bonferroni correction is therefore highly restrictive. The false positives are controlled at the expense of a loss in statistical power and hence the ability to discover true associations.

Tarone showed that, for discrete tests, a correction factor c≤m exists that guarantees control of the FWER using the concept of the minimum *p*-value. A hypothesis with minimum *p*-value above a significance threshold *δ* does not contribute to the FWER, as it cannot become significant; it is therefore *untestable*. Conversely, patterns with minimum *p*-value below *δ* are *testable* at that threshold. The correction factor *c* in Tarone’s procedure corresponds to the number of testable patterns and depends on the threshold, i.e. $\delta_{\text{tar}}=\alpha /c$, where *c* is a function of the current threshold *δ*. In practice, Tarone’s threshold can be computed efficiently using an iterative search strategy (Minato *et al.*, 2014) that subsequently lowers the threshold *δ* while enumerating patterns. We describe how this can be realized to mine segment interactions in Section 2.4.

Tarone’s procedure has been applied extensively in the field of computational biology for different problems and with different discrete tests (Bock *et al.*, 2018; Llinares-López *et al.*, 2015b, 2017; Minato *et al.*, 2014; Terada *et al.*, 2013). The extension to the CMH test (Llinares-López *et al.*, 2017; Papaxanthos *et al.*, 2016) led to a broader field of application, especially in the realm of genome-wide association studies where confounding factors such as population structure—if not properly addressed or corrected—can result in a large number of false positives.

#### 2.3.3 Westfall–Young permutations for FWER control

One disadvantage of Tarone’s procedure is its inability to correct for dependencies between tests. Interactions between gene segments are highly affected by the aforementioned sub- and super-pattern relationships (Section 2.2). Llinares-López *et al.* (2015a) developed a scalable version of the computationally demanding *Westfall–Young* permutations (Westfall *et al.*, 1993), called *Westfall–Young light*. The principle underlying permutation testing is the empirical estimation of the test statistic’s null distribution by permuting the class labels. This destroys all true association signals such that any significant association found is a false positive. Given a sufficient number of permutations *n_p_*, the FWER at a significance threshold *δ* can be estimated as

(1) $$\text{FWER}(\delta )=\frac{1}{n_{p}}\sum_{i=1}^{n_{p}}1(p_{min}^{i}\leq \delta ),$$

where 1(·) corresponds to the indicator function, and $p_{min}^{i}$ is the smallest *p*-value across all hypotheses for permutation *i*. The optimal threshold *δ_WY_* that controls the FWER at level *α* can be chosen as the lower *α*-quantile of $P={\left\{p_{min}^{i}\right\}}_{i=1}^{n_{p}}$. *Westfall–Young light* significantly improves the computational cost associated to permutation testing by incorporating Tarone’s testability criterion: since label permutations do not affect the minimum *p*-value, untestable patterns can be pruned from the search space, and their permutations can safely be skipped.

### 2.4 Our contributions: SiNIMin and SiNIMin-WY

In this section, we introduce our method SiNIMin and an extension based on Westfall–Young permutations (SiNIMin-WY). We provide a C++ implementation of both methods under https://github.com/BorgwardtLab/SiNIMin, including examples. Both methods use the concepts introduced in the previous sections to enable network-based mining of gene segment interactions and are based on the discrete CMH-test. Since they only differ in how they achieve control of the FWER, we start by explaining SiNIMin, and afterwards point out the modifications necessary for the SiNIMin-WY approach.

**Algorithm 1 SiNIMin approach**

**Input:** *n *×* d*-dimensional binary dataset **D**, phenotypes **y**, covariates **c**, edge-list E, target FWER *α*

**Output:** set of significant interactions S

1: **function**   main(D, c, y, E, α)

2:   Initialize **global**  ${\hat{\delta }}_{\text{tar}}\leftarrow 1$, **global**  $\hat{\alpha }\leftarrow 1$ and T←∅,

3:  P←  init_min_pvalues

4:  init_SiNIMinWY             ▹ only required for SiNIMin-WY

5:  **for**  $(g_{0},\;g_{1})\in E$  **do**

6:    $\mathrm{process}\_\mathrm{edge}(D,\;c,\;(g_{0},\;g_{1}))$

7:  **end for**

8:  $\delta^{*}\leftarrow \alpha /|T|$                   ▹ final significance threshold

9:  **return**  $\mathrm{filter}\_\mathrm{significant}(\delta^{*},\;y)$

10: **end function**

11:

12: **function**  $\mathrm{process}\_\mathrm{edge}(D,\;c,\;(g_{0},\;g_{1}))$

13:  $I^{\left(g_{0},g_{1}\right)}\leftarrow \mathrm{enumerate}\_\mathrm{segment}\_\mathrm{interactions}(g_{0},\;g_{1})$

14:  **for**  $I\in I^{\left(g_{0},g_{1}\right)}$  **do**

15:    Compute encoding $w_{I}$ for segment interaction I

16:    and the minimum *p*-value $p_{I}^{min}$ using D,c

17:    **if**  $p_{I}^{min}\leq {\hat{\delta }}_{\text{tar}}$  **then**

18:      T←T∪I

19:      process_SiNIMin(I)

20:    **end if**

21:    **if**  $\mathrm{is}\_\mathrm{prunable}(w_{I},\;c)$  **then**

22:      Remove all super-interactions from $I^{\left(g_{0},g_{1}\right)}$

23:    **end if**

24:  **end for**

25: **end function**

26:

27: **function**  process_SiNIMin(I)

28:  $\hat{\alpha }\leftarrow {\hat{\delta }}_{\text{tar}}\cdot |T|$

29:  **while**  $\hat{\alpha }\geq \alpha$  **do**

30:    ${\hat{\delta }}_{\text{tar}}\leftarrow$ next value from P, remove untestable patterns from T

31:    $\hat{\alpha }\leftarrow {\hat{\delta }}_{\text{tar}}\cdot |T|$

32:  **end while**

33: **end function**

34:

35: **function**  $\mathrm{filter}\_\mathrm{significant}(\delta^{*},\;y)$

36:  **return** interactions in T whose computed *p*-value $\leq \delta^{*}$

37: **end function**

#### 2.4.1 The SiNIMin method

SiNIMin enumerates all segment interactions in a depth-first strategy, by successively increasing the segment’s lengths. For every interaction, three crucial steps are performed: (i) assessment of testability by computing the minimum *p*-value, (ii) adjustment of the significance threshold $\hat{\delta_{\text{tar}}}$ to guarantee control of the FWER and (iii) potential pruning of super-patterns.

A high-level description of the method can be found in Algorithm 1. We refer the interested reader to the Supplementary Material, where a more detailed description of the method can be found. The method requires the binary data matrix **D**, a list of edges in form of gene-tuples E, the target FWER *α* (commonly α=0.05), the phenotype vector **y** and the covariate vector **c** as input. The output is a list of significant segment interactions S. The FWER estimate $\hat{\alpha }$ is set to 1 and the set of testable hypotheses T is initialized to the empty set, since no patterns have been explored yet. The significance threshold ${\hat{\delta }}_{\text{tar}}$ is initialized to 1, and it will be successively lowered to account for an increasing number of testable patterns found during the enumeration to guarantee control of the FWER. It will take on values from a list of decreasing, precomputed thresholds obtained by calling init_min_pvalues (Line 3). Lines 5–7 contain the heart of the algorithm: the call to process_edge, that tests all segments between the two genes and updates ${\hat{\delta }}_{\text{tar}}$. When invoking process_SiNIMin on edge (*g*_0_, *g*_1_), all segment interactions are enumerated and stored in $I^{\left(g_{0},g_{1}\right)}$. For every interaction in $I^{\left(g_{0},g_{1}\right)}$, we (i) compute its heterogeneity encoding $w_{I}$ and minimum *p*-value $p_{I}^{\min}$ and (ii) assess the testability (Line 17). In case a pattern is testable, it is stored in T and the function process_SiNIMin is invoked to ensure the FWER is controlled. An empirical FWER estimate can be obtained as ${\hat{\delta }}_{\text{tar}}\cdot |T|$ (Line 28). Whenever this value exceeds the given target *α*, the threshold ${\hat{\delta }}_{\text{tar}}$ is lowered until the FWER criterion is fulfilled (Lines 29–32). By lowering the threshold, previously testable patterns might become untestable and have to be removed from T. Notably, since ${\hat{\delta }}_{\text{tar}}$ decreases monotonically, untestable patterns with minimum *p*-value above ${\hat{\delta }}_{\text{tar}}$ can never become testable, thus guaranteeing control of the FWER. Lines 21–23 check whether there exist testable super-patterns of I. This can be inferred efficiently (Papaxanthos *et al.*, 2016), and constitutes a major increase in speed. Again, due to the monotonicity of ${\hat{\delta }}_{\text{tar}}$, we guarantee that prunable patterns remain prunable. Ultimately, after all edges have been processed, the final significance threshold $\delta^{*}$ is computed (Line 8) as α/|T|. Only now the phenotype **y** is used to infer *p*-values of all testable patterns in T, and significant patterns are returned.

#### 2.4.2 The SiNIMin-WY method

To incorporate Westfall–Young permutations, the algorithm has to be altered in three different places. First, in case of SiNIMin-WY, a method-specific initialization takes place (Algorithm 1, Line 4), during which the label permutations are generated, and an *n_p_*-dimensional vector of permutation *p*-values is initialized to 1 (Algorithm 2, Lines 1–6). It stores the smallest *p*-values across all *n_p_* permutations. Second, the FWER is estimated from the label permutations: To do so, we call process_SiNIMinWY instead of process_SiNIMin in Line 19. It uses the permuted labels to compute *n_p_* permutation *p*-values for the current interaction I (Algorithm 2, Lines 9–12). Those *p*-values falling below previously seen permutation *p*-values are stored, as they potentially correspond to newly occurring false positives (Algorithm 2, Line 11). After processing all permutations, the empirical FWER is estimated (see Eq. 1), and in case it exceeds the target value *α*, the significance threshold ${\hat{\delta }}_{\text{tar}}$ is lowered until the estimated FWER complies with *α*. The third change is the computation of the final significance threshold (Algorithm 1, Line 8). For SiNIMin-WY, we call threshold_SiNIMinWY instead. It computes the per-hypothesis significance threshold as the lower *α*-quantile of the vector $p_{\min}$. Since Westfall–Young permutations are computationally demanding, we parallelized the permutation testing using OpenMP.

**Algorithm 2 SiNIMin-WY specific functions**

1: **function**  init_SiNIMinWY

2:  **for**  $j=1,\;\ldots ,\;n_{p}$  **do**

3:    $y^{j}\leftarrow \mathrm{random}.\mathrm{permutation}(y)$

4:    $p_{\min}^{j}\leftarrow 1$

5:  **end for**

6: **end function**

7:

8: **function**  process_SiNIMinWY(I) 

9:  **for**  $j=1,\;\ldots ,\;n_{p}$  **do**

10:    Compute *p*-value $p_{I}^{j}$

11:    $p_{\min}^{i}=\min(p_{\min}^{i},p_{I}^{j})$

12:  **end for**

13:  **while**  $\text{FWER}({\hat{\delta }}_{\text{tar}})\leq \alpha$  **do**

14:    ${\hat{\delta }}_{\text{tar}}\leftarrow$ next value from P

15:  **end while**

16: **end function**

17:

18: **function**  threshold_SiNIMinWY

19:  **return** *α*-quantile of $p_{\min}$

20: **end function**

## 3 Simulation study

We assessed the performance of our methods SiNIMin and SiNIMin-WY to find statistically significant gene segment interactions under a model of genetic heterogeneity on simulated data. We compared our methods to several comparison partners and quantified performance in the form of runtime, statistical power and type-I error. Further analyses showing the usefulness of testing segment interactions under a model of genetic heterogeneity, as well as the robustness of our methods to network modifications can be found in Supplementary Section S2. The comparison partners are listed in Table 2, and can be categorized into six different groups (see columns) with respect to the types of feature-sets they test for association. A more detailed description can be found in the Supplementary Material.

**Table 2.** Overview of comparison partners

	SNPs	Genes	Edges	Segments	SNP-interactions	Segment-interactions
Tarone				FastCMH	edgeEpi (WY)	SiNIMin (WY)
FastLMM	FastLMM-singleSNP	FastLMM-genes	FastLMM-edge	FastLMM-segment		FastLMM-interact
SKAT-O		SKATO-genes	SKATO-edge	SKATO-segment		SKATO-interact
PLINK					PLINK-epistasis, PLINK-fast-epistasis	

*Note*: Columns indicate the features/feature-sets that were tested, rows the different method-classes that either fall into the FastLMM framework (Lippert *et al.*, 2011, 2014), the SKAT-O framework (Lee *et al.*, 2012), the Tarone framework (Llinares-López *et al.*, 2017) or the PLINK framework (Chang *et al.*, 2015) (see Supplementary Section S2.2 for details).

*Runtime analysis* We compared runtimes of the direct competitors of our proposed methods, i.e. all methods that test interactions between gene segments along edges in a network. We simulated artificial data (see Supplementary Material) and varied the two parameters that dominate the runtime: the number of variants per gene and the number of edges in the network. All methods were run on a high-performance computing cluster. We observed that SiNIMin and SiNIMin-WY clearly outperformed FastLMM-interact and SKATO-interact for all network and gene sizes (see Supplementary Fig. S4). Especially SKATO-interact is only applicable to small networks. See Supplementary Material for a more in-depth analysis of the runtimes.

**Fig. 2.**
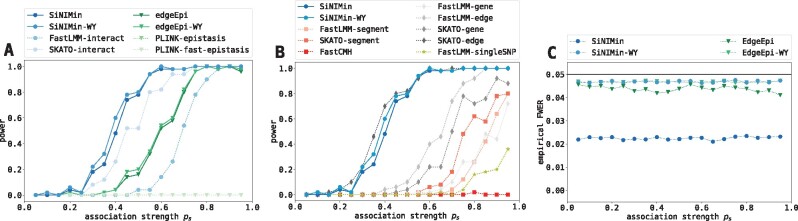
Power (1-type II error) and type I error analysis of the simulation study. We compared SiNIMin and SiNIMin-WY to (**A**) approaches that consider interactions between sets of genetic variants and (**B**) approaches that test sets of genetic variants (no interactions) (see Table 2 for comparison partners). Both figures show the power to detect the truly significant interaction for varying association strengths *p_s_*. (**C**) Empirical FWERs for varying association strengths *p_s_*. The black horizontal line indicates the target FWER (α=0.05)

*Power and FWER analysis* To evaluate the performance of SiNIMin and SiNIMin-WY, we generated artificial data with known ground truth. We created small networks containing 75 genes and 100 edges to allow an evaluation of *all* comparison partners (including SKATO-interact). Each dataset contained one *truly significant* gene segment interaction. We varied its association strength *p_s_* to the phenotype, with 1 indicating a very strong association (see Supplementary Material for details). To evaluate type II error, we compared our methods against the baselines with respect to power. Figure 2A and B shows power as a function of the association strength *p_s_* for interaction-based (either on the level of gene segments or individual genetic variants) and non-interaction-based methods, respectively. In general, we observed that interaction-based methods were better suited to detect the true association, except for the PLINK methods that are unable to handle the excessive number of tests (see Supplementary Fig. S2). Although SKATO-interact performed similar to SiNIMin and SiNIMin-WY, its application is limited to small datasets due to computational restrictions (see Supplementary Fig. S4). While SKATO-edge showed a performance comparable to SiNIMin and SiNIMin-WY, our proposed methods have the advantage of analyzing the data at a higher resolution: we can pinpoint the *variants* that drive the association, as opposed to the edge only. Next, we showed that our methods controlled the type I error by evaluating the empirical FWER (Fig. 2C). As for SiNIMin and SiNIMin-WY, we found that Westfall–Young permutations resulted in a less stringent control of the FWER. In summary, we showed that SiNIMin and SiNIMin-WY were able to detect the truly associated pattern, and outperformed the competitors in at least one of the following characteristics: (i) statistical power, (ii) runtime or (iii) resolution of association signal.

## 4 Analysis of *Arabidopsis thaliana* phenotypes

We applied our novel methods to a widely used *A.thaliana* GWAS dataset from the study by Atwell *et al.* (2010), downloaded from easyGWAS (Grimm *et al.*, 2017) and AraPheno (Seren *et al.*, 2017; Togninalli *et al.*, 2019).

*Experimental setup* We used genotype data for 20 dichotomous phenotypes (sample sizes ranging from 84 to 177, see Supplementary Table S2), and created categorical covariates with *k*-means, clustering the three leading eigenvectors of the empirical kinship matrix [following Llinares-López *et al.* (2017)]. The number of clusters *k* equaled the number covariate classes and was chosen such that the genomic inflation was as close to 1 as possible (see Supplementary Fig. S6). To represent interactions, we used the Interactome network (Consortium *et al.*, 2011), consisting of 11373 interactions between 4866 *A.thaliana* genes. We downloaded gene annotations from AraPort (Krishnakumar *et al.*, 2015), and represented each gene with SNPs that overlapped with its introns and exons. Out of the 4866 genes in the interactome network, we were able to represent 4431 genes with SNPs from the datasets (see Supplementary Material for details).

*Results* We applied our methods SiNIMin and SiNIMin-WY to the 20 *A.thaliana* phenotypes. We observed that including covariates into the analysis resulted in the desirable reduction of genomic inflation across 18 out of 20 phenotypes (Supplementary Fig. S6). Estimating the FWER using Tarone’s procedure increases power compared to the standard Bonferroni correction, as can be seen by the substantially lower number of testable patterns (see Supplementary Fig. S7 and Table S5). With SiNIMin-WY, we found significant gene segment interactions in 36 gene interactions, spanning 10 different phenotypes (Supplementary Files and Tables S3 and S6). We compared our approach against a variety of comparison partners and considered any gene interaction found by SiNIMin and SiNIMin-WY as *novel*, if none of the comparison partners detected one or both of the interacting genes. In total, SiNIMin-WY found nine novel gene interactions across seven different phenotypes (see Table 3). When restricting the segment length to 1 in SiNIMin-WY (yielding edgeEpi-WY), we detected two novel gene interactions spanning two additional phenotypes. Although those SNP interactions were also tested with SiNIMin-WY, they did not reach significance due to the larger number of tests, and the more stringent significance threshold. When comparing SiNIMin-WY to FastLMM-interact we found that SiNIMin-WY had a low false-negative rate, since only five interactions were missed (out of 25, Supplementary Table S4). We investigated the genes in Table 3 using the TAIR resource (Berardini *et al.*, 2015). All nine gene interactions contained genes that were either involved in similar biological processes, located in the same cellular components, shared molecular functions or were expressed in similar plant structures and expressed during similar developmental stages. The interaction AT1G15750-AT1G17380 was significant for the phenotypes *avrB* and *avrRpm1* (*p^avrB^* = 6.76e-08, $p^{\mathrm{avrRpm}1}$=1.15e-07), both are phenotypes of bacterial disease resistance. The two genes are involved in the *jasmonic acid-mediated signaling pathway*. Jasmonic acid mediates stress responses in plants (Delker *et al.*, 2006), and the gene AT1G17380 is known to be involved in the defense response and the response to wounding. Another interesting interaction was the one between AT5G25150 and AT5G45600 for the *Leafroll16* phenotype (* p* =* *3.89e-09). Both genes code for subunits of the General Transcription Factor IID (TFIID) in *A.thaliana* (Lawit *et al.*, 2007). The interaction between AT3G18490 and AT5G42980 for the *Leafroll22* phenotype (*p* =* *5.24e-09) linked two genes that show similar biological processes: AT3G18490 is involved in the response to water deprivation, and AT5G42980 is involved in the plant’s heat response. Other interactions contained related genes, for example the interaction AT4G16845–AT5G57380, where both genes are involved in the vernalization response in *A.thaliana*. However, the interaction’s connection to the phenotype *Hiks1*, a protist disease-resistance phenotype, remains to be uncovered.

**Table 3.** Novel hits for *A.thaliana*

Phenotype	Gene–gene interaction	*p*-value	SiNIMin-WY	SiNIMin	edgeEpi-WY	edgeEpi
avrB	AT1G15750–AT1G17380	6.76e-08	**2**	0	0	0
avrRpm1	AT1G15750–AT1G17380	1.15e-07	**2**	0	0	0
Chlorosis22	AT1G74490–AT2G41100	9.47e-08	**2**	0	0	0
Hiks1	AT1G15760–AT5G43460	2.19e-07	**10**	0	0	0
Hiks1	AT4G16845–AT5G57380	4.26e-08	**1**	0	0	0
Hiks1	AT4G19030–AT5G43460	2.19e-07	**2**	0	0	0
Leafroll16	AT5G25150–AT5G45600	3.89e-09	**23**	10	0	0
Leafroll22	AT3G18490–AT5G42980	5.24e-09	**16**	16	0	0
Noco2	AT2G01950–AT3G43850	1.75e-07	**2**	0	0	0.
avrRpt2	AT3G15660–AT4G15730	1.15e-07	0	0	**1**	1
Leafroll10	AT2G04630–AT3G56270	3.59e-07	0	0	**1**	0

*Note*: The last four columns contain the number of significant gene-segment interactions (SiNIMin, SiNIMin-WY) and SNP interactions (edgeEpi, edgeEpi-WY) in the novel hit. We report the lowest *p*-value for any pair of segments within the novel hit, and highlight the method in bold, for which this *p*-value is obtained. For more details on the significant segments, see Supplementary Table S6.

**Table 4.** Novel hits for migraine cohorts

Dataset	Gene-interaction	*p-value*	SiNIMin-WY	SiNIMin	edgeEpi-WY	edgeEpi
dMaMo	EPHA6–TIAM1	2.64e-08	**1**	0	**1**	1
gMaMo	BMP4–BMPR1B	1.50e-07	0	0	**1**	0
gMaMo	HAO1–VDAC3	1.33e-07	0	0	**2**	0

*Note*: The last four columns contain the number of significant gene segment interactions (SiNIMin, SiNIMin-WY) and SNP interactions (edgeEpi, edgeEpi-WY) in the novel hit. We report the lowest *p*-value for any pair of segments within the novel hit, and highlight the method in bold, for which this *p*-value is obtained. For more details on the significant segments, see Supplementary Table S10.

## 5 Study on low-frequency variants in migraine

As a second application, we aimed to improve our understanding of the genetics underlying two migraine subtypes, namely migraine with aura and migraine without aura. Patients that suffer from the first subtype experience visual disturbances before onset of other migraine symptoms. In this study, we restricted the dataset to low-frequency variants, that is SNPs with a minor allele frequency below 5%. Note that, combinations of gene segments could still achieve frequencies above 5%.

*Experimental setup* We pooled data from five different migraine cohorts: a Dutch cohort with aura (DMA), a Dutch cohort without aura (DMO), a German cohort with aura (GMA), a German cohort without aura (GMO) and a Finnish cohort with aura (FMA). Cases from the five cohorts were combined, and a binary phenotype was created such that migraine patients with aura obtained label 1. This constituted the *MaMo* cohort, comprising 5013 samples. We repeated this procedure, and this time combined migraine patients with the same nationality, which resulted in one dataset of 1849 Dutch patients (*dMaMo*) and one of 2231 German patients (*gMaMo*). Covariates were inferred from the genetic relationship matrix (see Supplementary Material). We used the InBio network (Li *et al.*, 2017) to derive interactions, and mapped SNPs to genes that fell within a 50 kb window up- and downstream of the genes (see Supplementary Table S7 and Fig. S8).

*Results* All three cohorts exhibited inflation ($\lambda_{gc}^{\mathrm{MaMo}}=4.85$, $\lambda_{gc}^{\mathrm{gMaMo}}=1.19,\;\lambda_{gc}^{\mathrm{dMaMo}}=1.21$ with SiNIMin-WY) if no covariates were used. On including covariates, we observed a reduction in inflation for all cohorts (see Supplementary Figs S9–S12). SiNIMin-WY detected a total of 1304 significant gene segment interactions in MaMo, 737 in gMaMo and 1126 in dMaMo (Supplementary Table S8). Out of those, one interaction in the dMaMo cohort was novel, between the EPHA6 and TIAM1 (*p *=* *2.64e-08) genes, two genes that are involved in ephrin signaling. The ephrin-B signaling pathway has been previously associated with migraine (Guyuron *et al.*, 2014). Notably, this is an interaction between length-1 segments, and hence could also be detected with edgeEpi-WY. Restricting the segment length to 1 (edgeEpi-WY), two more novel interactions were detected (see Table 4), one of them between the BMP4 and BMPR1B genes (*p *=* *1.50e-07). They interact within the BMP signaling pathway, which in turn has been linked to neural development (Bond *et al.*, 2012). The third novel gene interaction (HAO1–VDAC3, *p *=* *1.33e-07), was between two genes whose edge had a low-confidence (score 0.247) in the InBio network. It was not part of any of the underlying pathway or interaction data bases the InBio network was built on (e.g. because it might have been inferred by orthology), and hence, we could not deduct any biological interpretation. However, reporting edges that showed no strong evidence indicated the potential of our methods to provide further support for low-confidence interactions in networks, and might mark the starting point for additional experiments.

## 6 Discussion and conclusions

We introduce two novel methods, SiNIMin and its Westfall–Young permutation-based counterpart SiNIMin-WY (Westfall *et al.*, 1993), to detect pairwise interactions between gene segments that are associated with a binary phenotype under a model of genetic heterogeneity. We guide our search for interactions using biological prior knowledge in the form of PPI networks and restrict the search space to interactions between gene segments that fall into genes connected by an edge. To enable this computationally and statistically challenging search for interactions, our methods are based on established methods in the field of significant pattern mining. This reformulation requires categorical data, and non-categorical data have to be preprocessed prior to the application of SiNIMin. While this might seem as a limitation, it permits exploring the enormous search space associated with mining gene segment interactions, a task that cannot be achieved with other methods. Using other statistical tests, for example likelihood ratio tests, requires the exploration of all possible segment interactions between adjacent genes, which is prohibitive from a computational and a statistical point of view, due to the intrinsic multiple hypothesis problem. We evaluated the performance of our method on simulated datasets and showed that it was well-powered to detect true associations while controlling the type I error. Application of our methods to *A.thaliana* and migraine data showed their capability to find novel interactions between genes that discriminate cases and controls.

In contrast to methods that rely on estimating joint effects of markers based on the marker’s marginal statistics (e.g. Azencott *et al.*, 2013; Reyna *et al.*, 2018), our approach is not restricted to detecting significant interactions in which at least one of the interaction partners shows marginal associations. By combining the gene segments according to a genetic heterogeneity model prior to the statistical test, our approach is capable of detecting interactions that do *not* exhibit a marginal signal in any of the interaction partners. However, for increasing dataset sizes, our method scales with the number of genetic variants that are mapped to genes, i.e. the computational and statistical burden increases, which affects the applied set-based tests (Lee *et al.*, 2012; Lippert *et al.*, 2014) to a lesser extent, as they scale with the number of genes or edges in a network.

An interesting direction of future research is to deviate from the strict mining of interactions between pairs of genes, which could be achieved by taking larger groups of interacting genes into account and focusing on network modules (see e.g. Mezlini and Goldenberg, 2017; Reyna *et al.*, 2018). Moreover, recent advances in the field of frequent itemset mining (e.g. Fowkes and Sutton, 2016) could be integrated into our approach to find representative patterns of the phenotypic classes and test those, instead of exploring all segment interactions.

## Funding

This work was supported by the SNSF Starting Grant ‘Significant Pattern Mining’ (no. 155913, K.B.) and in part by the Alfried Krupp Prize for Young University Teachers of the Alfried Krupp von Bohlen und Halbach-Stiftung (K.B.).

*Conflict of Interest*: none declared.

## Supplementary Material

btaa581_Supplementary_Data
