# Supplementary material for: Network-guided search for genetic heterogeneity between gene pairs
Source: Bioinformatics. 2020 Jun 23;37(1):57–65. doi: 10.1093/bioinformatics/btaa581 (PMC8034561; doi:10.1093/bioinformatics/btaa581)
Supplement: btaa581_Supplementary_Data [file btaa581_supplementary_data.pdf]

Supplementary Material

Network-guided search for genetic heterogeneity between gene pairs

Anja C. Gumpinger, Bastian Rieck, Dominik Grimm,
International Headache Genetics Consortium and Karsten Borgwardt

Contents

S1 Method **S2**
S1.1 Extended algorithm S2
S1.2 Computational complexity S3

S2 Simulation Study **S5**
S2.1 Simulation of artificial data S5
S2.2 Baseline methods S6
S2.3 Effect of network versus effect of heterogeneity model S7
S2.4 Effect of network manipulations S8
S2.5 Runtime analysis S9

S3 Study of *Arabidopsis thaliana* **S10**
S3.1 Data preprocessing S10
S3.2 Control of genomic inflation S10
S3.3 Results S11

S4 Study of migraine subtypes **S18**
S4.1 Data preprocessing S18
S4.2 Control of genomic inflation S18
S4.3 Results S19
S4.4 Analysis of gender covariates S21

S1 Method

S1.1 Extended algorithm

In this section we provide a more comprehensive description of the algorithm explained in the main text of the paper. We provide pseudocode for the `SiNIMin` method (see Alg. 1 and 2). All explanations are added as comments *inline*. For the `SiNIMin-WY` approach, the code in Alg. 1 has to be altered at only three positions, namely the initialization (Line 4), the estimation of the empirical family-wise error rate (Line 24), and the computation of the final significance threshold (Line 8). Details on the modifications are already described in the main paper, and apply here in exact the same way. Hence, we refrain from explicitly explaining the `SiNIMin-WY` approach a second time, but indicate the modifications in the comments. For the modifications, we refer the reader to the main text.

Algorithm 1 `SiNIMin`

Input: $n \times d$-dimensional binary dataset $\mathbf{D}$, phenotypes $\mathbf{y}$, covariates $\mathbf{c}$, edge-list $\mathcal{E}$, target FWER α,
Output: set of significant interactions $\mathcal{S}$

1: **function** MAIN($\mathbf{D}, \mathbf{c}, \mathbf{y}, \mathcal{E}, \alpha$)
2: Initialize **global** $\widehat{\delta}_{\text{tar}} \leftarrow 1$, **global** $\widehat{\alpha} \leftarrow 1$ and $\mathcal{T} \leftarrow \emptyset$,
3: $\mathcal{P} \leftarrow$ INIT_MIN_PVALUES( ) ▷ initialization of p-value thresholds for $\widehat{\delta}_{\text{tar}}$
4: INIT_SINIMIN_WY( ) ▷ additional initialization only required for `SiNIMin-WY`
5: **for** $(g_0, g_1) \in \mathcal{E}$ **do**
6: PROCESS_EDGE((g_0, g_1)) ▷ processing of all segment interactions in edge
7: **end for**
8: $\delta^* \leftarrow \alpha/|\mathcal{T}|$ ▷ computation of per-hypothesis threshold, adapt for `SiNIMin-WY`
9: **return** FILTER_SIGNIFICANT($\delta^*, \mathcal{T}, \mathbf{y}$) ▷ reporting of significant segment interactions
10: **end function**
11:
12: **function** PROCESS_EDGE($\mathbf{D}, (g_0, g_1)$)
13: g_0.segments $\leftarrow$ ENUMERATE_ALL_SEGMENTS(g_0) ▷ Enumeration of all segments in gene g_0
14: **while** g_0.segments **do**
15: g_0.current $\leftarrow$ g_0.segments.`pop()` ▷ iterate all segments in g_0, and for the current one...
16: **for** $s_1 \in \{g_1.\text{start}, ..., g_1.\text{end}\}$ **do**
17: $l_1 = 0$
18: **while** $l_1 \leq g_1.\text{end} - g_1.\text{start}$ **do**
19: $l_1 \leftarrow l_1 + 1$ ▷ ... enumerate all segments in interacting gene g_1
20: Compute encoding $\mathbf{w}_{\mathcal{I}}$ between segments g_0.current
21: and (s_1, l_1) and the minimum p-value $p_{\mathcal{I}}^{\min}$ using $\mathbf{D}, \mathbf{c}$
22: **if** $p_{\mathcal{I}}^{\min} \leq \widehat{\delta}_{\text{tar}}$ **then** ▷ assess testability
23: $\mathcal{T} \leftarrow \mathcal{T} \cup \mathcal{I}$ ▷ add to set of testables
24: PROCESS_INTERACTION($\mathcal{I}$) ▷ update FWER criterion, adapt for `SiNIMin-WY`
25: **end if**
26: **if** IS_PRUNABLE($\mathbf{w}_{\mathcal{I}}, \mathbf{c}$) **then** ▷ mark all super-patterns as untestable and do not process them
27: **break**
28: **end if**
29: **end while**
30: **end for**
31: **end while**
32: **end function**

Algorithm 2 Auxiliary functions

```
function INIT_MIN_PVALUES
    Precompute a list 𝒫 of decreasing p-values that will
    be attained by δ̂_tar to guarantee control of the FWER.
    return 𝒫
end function

function ENUMERATE_ALL_SEGMENTS(g_i)
    segments ← ∅
    for s_1 ∈ {g_i.start, ..., g_i.end} do
        l_i = 0
        while l_1 ≤ g_i.end − g_i.start do
            l_i ← l_i + 1
            segments.push_back(s_i, l_i)
        end while
    end for
    return segments
end function

function PROCESS_INTERACTION(ℐ)
    α̂ ← δ̂_tar · |𝒯|
    while α̂ ≥ α do
        δ̂_tar ← next value from 𝒫, remove untestable patterns from 𝒯
        α̂ ← δ̂_tar · |𝒯|
    end while
end function

function IS_PRUNABLE(w, c)
    Compute the minimum p-value p^s of all super-patterns
    of the current pattern ℐ
    if p^s > δ̂_tar then
        return True
    else
        return False
    end if
end function

function FILTER_SIGNIFICANT(δ*, 𝒯, y)
    Compute the p-value for all interactions in 𝒯
    return interactions in 𝒯 with p-value ≤ δ*
end function
```

S1.2 Computational complexity

To derive the *computational complexity* (in terms of Landau symbols) of our method we have to consider that the method scales with the number of interactions in the network. The number of interactions in turn depends on the number of edges in the network, the number of segments per node, and the connectivity of the network. Let us assume a data set consisting of d features and a network with n nodes and m edges, and let us denote the total number of interactions with c. Let us start by first estimating the best and worst-case scenario per edge.

S1.2.1 Number of interactions per edge

Assume that the network just contains one edge between two nodes. All d features are mapped to the two nodes, i.e. one node is represented by d_1 features, the second one by d_2 features, and $d = d_1 + d_2$. In general, if a node is represented by d_1 features, there exists a total of $\frac{1}{2}d_1(d_1+1)$ segments within the node. The edge between the two nodes, the number of possible interactions c_e is therefore

$$\begin{aligned} c_e &= \frac{1}{2}d_1(d_1+1) \times \frac{1}{2}d_2(d_2+1) \\ &= \frac{1}{4}(d_1^2 + d_1)(d_2^2 + d_2). \end{aligned} \tag{1}$$

We want to infer the distribution of features to nodes that maximizes and minimizes the number of segment interactions between the nodes, while keeping the number of features mapped to the nodes unchanged. Let us first consider the case where the features are uniformly distributed across the nodes, that is $d_1 = d_2 = \frac{d}{2}$. Then Eq. 1 becomes:

$$\begin{aligned} c_e &= \frac{1}{4}\left(\frac{d^2}{4} + \frac{d}{2}\right)^2 \\ &= \frac{1}{4}\left(\frac{d^4}{16} + \frac{d^3}{4} + \frac{d^2}{4}\right) \end{aligned} \tag{2}$$

Next, we consider the case where the features are not distributed uniformly, but there is an offset of size a, that is $d_1 = \frac{d}{2} + a$, $d_2 = \frac{d}{2} - a$, and $0 < a < \frac{d}{2}$. In this case, the number of interactions between the two nodes becomes

$$\begin{aligned} c_e &= \frac{1}{4}\left(\left(\frac{d}{2} + a\right)^2 + \frac{d}{2} + a\right)\left(\left(\frac{d}{2} - a\right)^2 + \frac{d}{2} - a\right) \\ &= \frac{1}{4}\left(\frac{1}{16}d^4 + \frac{1}{4}d^3 + \frac{1 - 2a^2}{4}d^2 - a^2 d + a^4 - a^2\right) \end{aligned} \tag{3}$$

We can show that $d_1 = d_2 = \frac{d}{2}$ maximizes the number of interactions between the nodes by showing that Eq. 3 is always smaller than Eq. 2. This results in the inequality

$$-\frac{1}{2}a^2 d^2 - a^2 d - a^2 + a^4 \leq 0,$$

which is always the case for $0 < a < \frac{d}{2}$. We can show that the above Eq. 3 has two more roots in a, corresponding to the value of a that gives the minimum number of interactions.

$$a_{1,2} = \pm\frac{1}{2}\sqrt{(d-1)^2 - 3} \tag{4}$$

This is a real-valued root, and the integer closest to the root is $a_1 = \frac{d}{2} - 1$, which corresponds to the value of a that results in the lowest number of segment interactions between the two nodes. In other words, if all but one features are mapped to the first node, and the remaining feature is mapped to the second one, the number of interactions is minimized. All together, the worst case complexity for one edge can be written as $\mathcal{O}(d^4)$.

S1.2.2 Number of interactions across data set

The findings from the previous section can now be translated to the complete network. Assuming a total of d features, the worst case number of interactions per-edge is reached if all genes have the same number of features assigned to them. Hence, the number of interactions to test is

$$c = \frac{1}{4}m\left(\frac{d^4}{n^2} + 2\frac{d^3}{n^3} + \frac{d^2}{n^2}\right), \tag{5}$$

i.e. the runtime complexity can be written as $\mathcal{O}\left(m\frac{d^4}{n^4}\right)$. Additionally, assuming that the graph is fully connected, the number of edges m can be written as $m = n(n-1)$, and the complexity becomes. $\mathcal{O}\left(\frac{d^4}{n^2}\right)$.

S1.2.3 Evaluation for real data sets

We empirically compute the number of interactions using the above formula Eq. 5 for the real data sets used later, i.e. the *A. thaliana* and the three migraine cohorts. The results are listed in Tab. S1. This demonstrates that the bound is indeed a worst-case scenario that 'dominates' the empirical number of interactions. Finding a more precise bound, while desirable, is left for future work and beyond the scope of this paper.

Table S1: Worst case number of gene segment interactions for the data sets used later in the paper, namely the *A. thaliana* and migraine data sets.

dataset	worst-case	observed
A.thaliana	43,534,211,553,865	6,617,466
MaMo	20,631,753,391,952	8,743,261
dMaMo	22,171,027,746,983	9,133,634
gMaMo	20,512,892,033,990	8,502,931

S2 Simulation Study

S2.1 Simulation of artificial data

The simulation follows the one described in [1]. We generate artificial data for a randomly generated network with 75 nodes (representing the genes) and 100 edges (representing the interactions between genes). In each simulation, we generate data for 500 samples. For each of the genes g_i, $i \in [1, 75]$, we simulate a random number n_{g_i} of SNPs that are mapped to gene g_i. We draw n_{g_i} from a uniform distribution over the range $[1, 10]$. We furthermore allow genes to overlap with a probability of 30%, where the extend of overlap is limited to at most half of the SNPs per gene. This results in a varying number of features per data set: $d = \sum_{i=1}^{75} n_{g_i}$, where $75 \leq d \leq 750$, since $1 \leq n_{g_i} \leq 10$.

S2.1.1 Simulation of data matrix containing a significant and confounded segment interaction

We simulate a binary data matrix $\mathbf{D} \in [0, 1]^{d \times 500}$ at random, where the probability of observing a 1 at any position equals 0.3. For every simulated data set, we generate two segment interactions, a truly significant interaction, and a confounded interaction. Both interactions come from the combination of two segments, with the segments inserted into two interacting genes (that is an edge). For both, the significant and the confounded interaction, we fix the combined length of both segments to six SNPs, and draw the length of each individual segment at random, with a minimum size of 2, and a maximum size of 3 SNPs per segment. In other words, each significant/confounded interaction is the combination of two segments (s_0, l_0) and $(s_1, 6-l_0)$, where $l_0 \sim \mathcal{U}([2, 3])$. To simulate the binary data for the truly significant and confounded interaction, we proceed as follows: We start by simulating three binary vectors

1. the binary phenotype vector $\mathbf{y}$
2. the encoding of the truly significant interaction $\mathbf{e_s}$
3. the encoding of the confounded interaction $\mathbf{e_c}$

from a multivariate Binomial distribution with mean vector μ and covariance matrix Σ (using the R-package `binsim`), where

$$\mu = \begin{bmatrix} 0.5 & 0.5 & 0.5 \end{bmatrix}, \quad \Sigma = \begin{bmatrix} 1 & p_s/2 & p_{con}/2 \\ p_s/2 & 1 & 0 \\ p_{con}/2 & 0 & 1 \end{bmatrix}.$$

The parameters p_s and p_{con} regulate the strength of the association signal between the phenotype and the significant and confounded interaction, respectively. For all simulations, we set $p_s = p_{con}$. The process

of data generation is equivalent for the truly associated interaction $\mathbf{I}$ and the confounded interaction $\mathbf{C}$, which is why we resort to only explaining the simulation of $\mathbf{I}$.
We need to expand the encoding vector $\mathbf{e_s}$, to result in a 6×500 matrix $\mathbf{I} \in [0,1]^{10\times 500}$ that will be inserted into the data matrix $\mathbf{D}$ at the position of the SNPs that are part of the significant interaction. When expanding the encoding vector, we guarantee that for every sample i with $\mathbf{e_s}[i] = 1$, exactly one of the ten SNPs gets assigned a 1. In this way, we minimize the association signal of individual SNPs in the interaction.
Next, we randomly draw an edge from the network, and chose the lengths and starting positions of the SNPs in the interacting segments. We split the matrix $\mathbf{I}$ according to the segment lengths, and insert the resulting two parts into the data matrix.
The same procedure is used to insert the confounded interaction into the data set. When choosing an edge, we make sure that none of the SNPs overlap with any of the SNPs in the truly significant interaction.

S2.1.2 Generation of covariate vector c

Following [1], we add a small disturbance to the confounding vector $\mathbf{e_c}$ by flipping each of its values with probability $p_\epsilon = 0.05$, resulting in the covariate vector $\mathbf{c}$.

S2.2 Baseline methods

In the following, we briefly describe the comparison partners used in our analysis, and the way in which we use them. For all of the baseline-methods we use gene-only data, i.e. all SNPs that are not mapped to a gene are removed from the data set prior to analysis. Note that this implies that any SNP can be mapped to at least one gene. This is done to guarantee a fair comparison between our proposed methods, and the baselines.

In general, we test the following types of SNP sets: (i) single SNPs, (ii) pairwise SNP interactions, (iii) all SNPs in a gene, (iv) all SNPs in an edge, (v) consecutive segments of SNPs and (vi) interactions between SNP segments. To test the SNP sets for association, we apply different methods, namely mixed-model approaches using the FastLMM framework, the SKAT-framework, as well as methods based on Tarone's procedure. Additionally, we apply an Epistasis screen using the PLINK framework. See also Tab. 1 in the main text for an overview. Note that not all baselines could be run on all settings.

S2.2.1 Univariate FastLMM

FastLMM [2] implements a linear mixed model approach to test univariate SNPs for association with a phenotype. We use the python2 package `fastlmm`. We apply FastLMM to test individual SNPs for their association with the phenotype, and report them together with the genes that contain the significant SNPs. We correct for MHT with a classical Bonferroni correction at significance level $\alpha = 0.05$.

S2.2.2 Set-based FastLMM

Similar to FastLMM, the set-based approach implements a linear mixed model approach to test groups of SNPs for joint association with a phenotype [3]. We test SNP-sets (iii)-(vi) listed above for joint association to the binary phenotype. FastLMM includes information on relatedness between samples into the model to account for population structure. In order to avoid proximal contamination, i.e. that the SNPs tested are also used to estimate the relationships, we ensure that no SNPs from the set to test are used to infer relatedness. We use the score-test and correct for MHT with a Bonferroni correction at significance level $\alpha = 0.05$. Any significant hit is reported, together with the gene or edge in the network

it originates from. Note that set-based FastLMM requires the enumeration of all sets to test prior to execution of the testing. This is a bottleneck if the number of sets to enumerate is large, as is the case for the enumeration of segment interactions.

S2.2.3 SKAT-O

SKAT-O [4] is a test developed for the analysis of rare variants, and tests groups of SNPs for their association with a phenotype. We use the R-package `SKAT`. Similar to FastLMM, we test SNP-sets (iii)-(vi) listed above for joint association to the binary phenotype with the SKAT-O test. We include covariates into the model as the c leading eigenvectors of the kinship matrix. We let c vary between 0 and 3, and chose the number of covariates that resulted in the least inflation/deflation of test statistics, as measured by the genomic inflation factor λ_{GC}. To correct for MHT, SKAT-O computes the number of effective tests to correct for dependencies among test. To determine the significant hits, we use this number of effective tests as correction factor to control the FWER, i.e. $\delta = \alpha/n_{eff}$, where n_{eff} corresponds to the number of effective tests, and $\alpha = 0.05$.

S2.2.4 FastCMH

FastCMH [1] is a method to detect segments of SNPs that are associated to a phenotype of interest under a model of genetic heterogeneity. It is based on Tarone's procedure to control the FWER, and also uses the CMH test, such that covariates can be integrated. The tested entities in FastCMH correspond to consecutive segments of SNPs, that do *not* use any notation of genes or edges. Note that this implies that segments are tested that might contain SNPs from multiple genes. For every significant segment, we report it along with the set of genes that contain any of the SNPs in the segment.

S2.2.5 PLINK - epistasis

We use PLINK to test for epistatic effects between SNPs using the commandline tool with the `--epistasis` and `--fast-epistasis` flags [5]. Note that this tests effects between any two SNPs in the data set, and is not restricted to SNPs coming from interacting genes, such as the `edgeEpi`-based methods. We control the FWER with the classical Bonferroni correction at significance level $\alpha = 0.05$.

S2.3 Effect of network versus effect of heterogeneity model

In order to validate whether the improvement in performance is due to the heterogeneity encoding or the combination of segments, or a combination of both, we compare several methods simulated data. Precisely, we simulated data as described in Section S2.1, assuming that the true association follows the heterogeneity model.

S2.3.1 Heterogeneity encoding vs. random effects model

We compare our proposed methods that operate on (a) segment interations (`SiNIMin`), (b) segments (without interactions) and (c) single SNPs. For all three types of patterns, we compare methods that rely on our heterogeneity encoding combined with Tarone's procedure, and conventional methods that apply random effect models to the SNPs in those patterns (see Fig. S1).

We observe that for all pattern types the combination of the binary heterogeneity encoding with Tarone's procedure outperforms the baselines in our simulation setting. In general, a major advantage of our proposed methods towards the random effects models that potentially explains this improvement is the capability to deem hypothesis untestable, thereby reducing the multiple hypothesis testing burden inherent to testing large numbers of patterns simultaneously.

(a) Segment interactions **(b)** Segments **(c)** Univariate SNPs

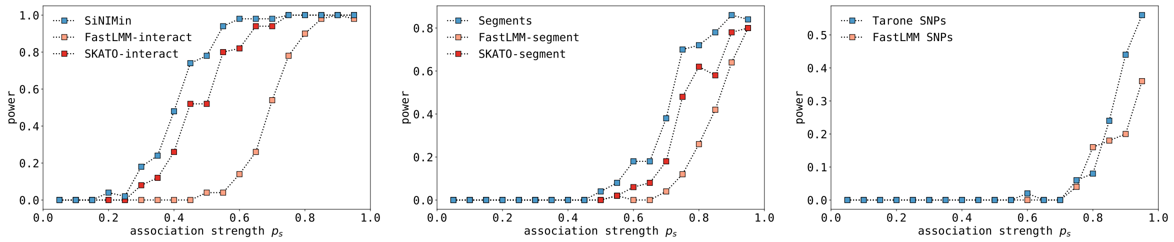

Figure S1: Comparison of different methods using the proposed heterogeneity encoding (in blue), compared to a standard encoding (in red colors) as used by FastLMM and SKAT-O. (a) segment-interaction methods, (b) segment methods, (c) univariate SNPs.

However, one should be aware of the different hypothesis underlying the random effects model and the proposed heterogeneity encoding. The heterogeneity model assumes a correlation between the presence of any minor allele and the phenotype, while the random effects model assumes that the variation in phenotype can be partially explained by the variance in genetics.

S2.3.2 Effect of interactions

In order to evaluate the benefit of testing interactions, we compare different approaches that all rely on a binary heterogeneity encoding combined with Tarone's procedure. We consider interactions at different scales and evaluate the impact of network-guided interaction search. We report the power of the following methods:

1. testing individual gene segments (`Segments`)
2. testing interactions between segments along edges in a network (`SiNIMin`)
3. testing all possible interactions between segments (`SiNIMin-full`)

We repeat the same analyses on the SNP level, that is we test the following patterns:

1. testing individual SNPs (`SNP`)
2. testing interactions between SNPs along edges in a network (`edgeEpi`)
3. testing all possible interactions between SNPs (`edgeEpi-full`)

The results of this analysis are illustrated in Fig. S2. We observe that, in general, methods based on interactions always outperform their univariate counterparts, that is testing of segments or individual SNPs. We find that testing interactions along the network increases power compared to testing all possible interactions (which is equivalent to using a fully connected network). This is due to the increase of tests in the fully-connected setting, resulting in an increasing burden of multiple hypothesis testing.

S2.4 Effect of network manipulations

Biological networks, especially protein-protein interaction networks, are well known to be noisy: due to the multiple sources that might give rise to a PPI, some edges might be false-positives, and many edges might not be discovered yet. Our methods `SiNIMin` and `SiNIMin-WY` treat the underlying network as given, that is only patterns along the network are explored. We test the effect of two types of manipulations to the network in a simulation study. (1) random removal of edges, and (2) random addition of edges to the network. For both cases, we remove/add between 5 and 50% of the edges and run `SiNIMin` on the data sets with manipulated networks. We observe that the random removal of edges has a significant effect on the power of our method to detect the true positives (Fig. S3a). This is due to the fact that, if the true positive lies in an edge that is removed, the corresponding hypothesis is not

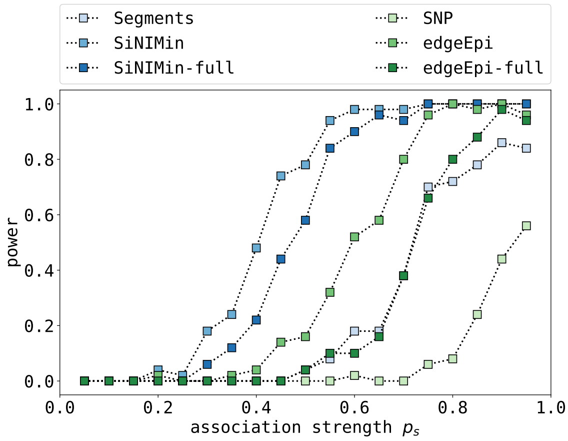

Figure S2: Power of methods based on gene segments (blue), and SNPs (green)

(a) random edges removed **(b)** random edges added

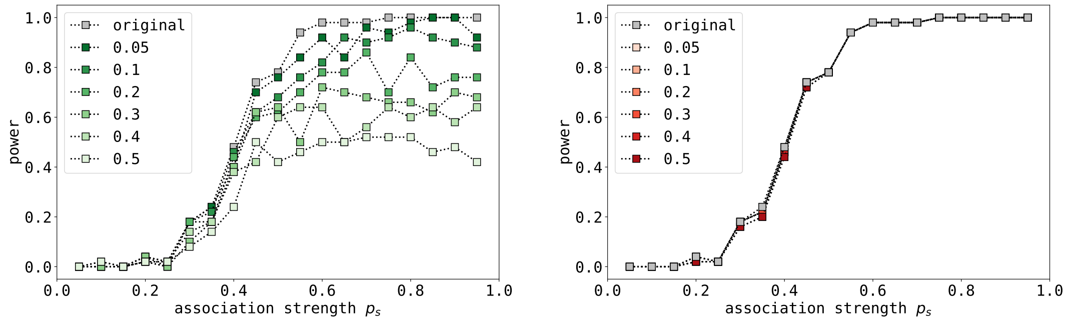

Figure S3: Effect of randomly removing (a) and adding (b) varying percentages of edges from the network.

tested. Thus, observing the drop of power to approx. 0.5 when 50% of edges are removed is expected, as in 50% of simulations, the corresponding edge will be removed. Inversely, adding random edges to the network does not have a significant effect on the power of our method, as the true positives are still contained in the space of hypothesis (Fig. S3b). One should note that this is a small simulation study, and if the number of edges is increased, we will observe a drop in performance caused by the increased number of hypothesis due to new edges. An extreme case of this can be observed with `SiNIMin-full` in Fig. S2, where a fully connected network is considered.

S2.5 Runtime analysis

We compare the runtimes of the different methods based on testing segment interactions within interacting genes, i.e. exactly the sets our methods `SiNIMin` and `SiNIMin-WY` test. For `SKATO-interact` and `FastLMM-interact` this requires the exhaustive enumeration of *all* such interactions, which is a time and memory consuming task (not counted towards runtime), while our proposed methods do this enumeration step on the fly. The empirical runtimes obtained when executing all methods on a high-performance computing cluster are illustrated in Fig. S4. The runtimes are affected by the number of segment interactions to test, which in turn are determined by the number of genetic variants n_s per gene, as well as the number of edges n_e in the network. We vary those two parameters $n_s \in \{5, 10, 15, 25, 35, 50\}$ and $n_e \in \{10, 25, 50, 100, 250, 500, 1000\}$. We set 120 hours as a time-cutoff (indicated by red line).

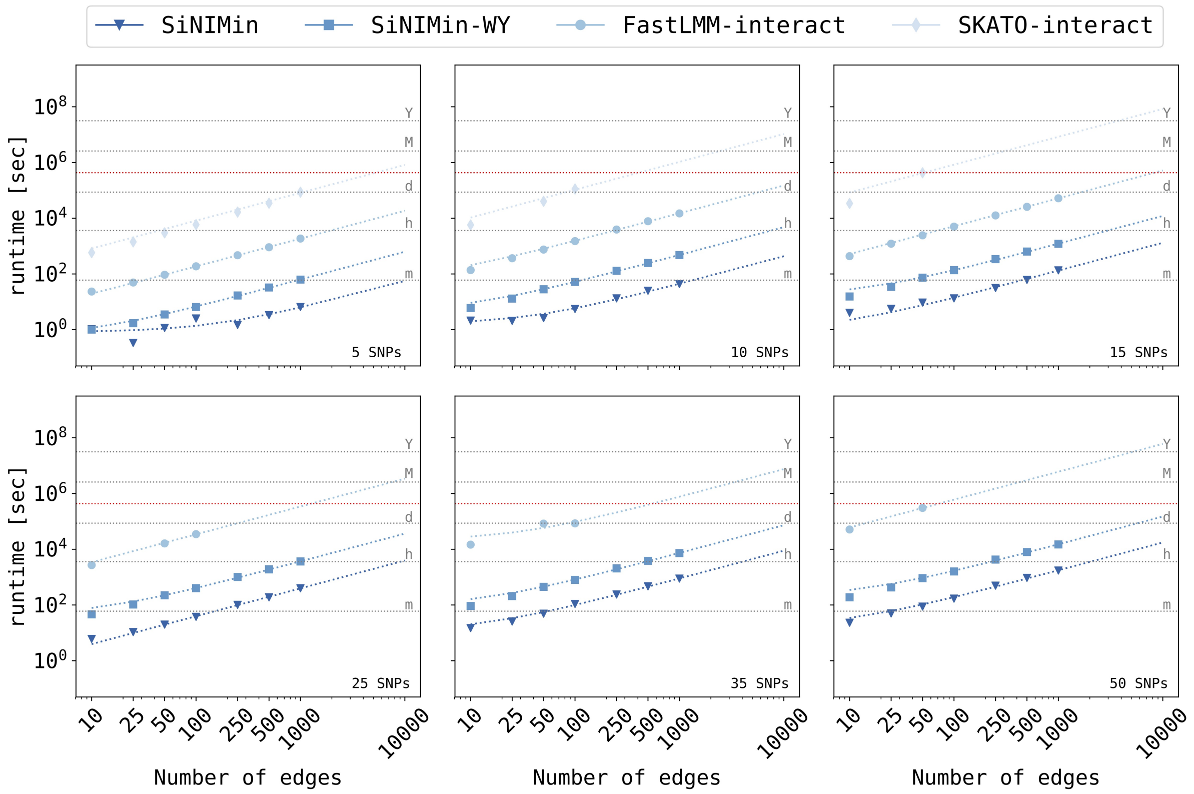

Figure S4: Runtimes of different segment interactions methods for varying data set sizes, depending on the number of genetic variants (SNPs) per gene and the number of edges in the network. Grey horizontal lines indicate time-scales, where the following abbreviations are used: m (1 minute), h (1 hour), d (1 day), M (1 month), Y (1 year). The red line indicates 120 hours, which is the maximum runtime we allow in this experiment. The data points correspond to averages over 10 runs on a high-performance computing cluster. The dashed lines correspond to linear fits through the data points and are supposed to indicate measurements for those data points that could not be tested empirically.

S3 Study of *Arabidopsis thaliana*

S3.1 Data preprocessing

The data set consists of a total of 214,051 SNPs. In total, there exist 107 phenotypes, out of which 21 are dichotomous. We omit the binary YEL phenotype due to its large class imbalance, and use the remaining 20. For each phenotype, the data set contains between between 84 and 177 accessions (Tab. S2). To represent interactions, we use the Interactome network [6], consisting of 11,373 interactions between 4,866 *A.thaliana* genes. We downloaded gene annotations from AraPort [7], and represent each gene with SNPs that fall into its coding region. Out of the 4,866 genes in the interactome network, we were able to represent 4,431 genes with SNPs from the data sets. In total, 24,571 SNPs are mapped to genes (Fig. S5).

S3.2 Control of genomic inflation

To reduce the impact of population structure, we create categorical covariates for each data set separately by clustering the three leading eigenvectors of the empirical kinship matrix. Clusters are inferred with *k-means*, and the number of clusters k corresponds to the number of categorical covariates. For our

Table S2: Description of the 20 dichotomous phenotypes of the *A. thaliana* data set from [8] used in the analysis. The binary *YEL* phenotype was not used due to large class imbalance.

phenotype	trait ontology (from [9])	samples	cases
Anthocyanin10	anthocyanin content	177	33
Anthocyanin16	anthocyanin content	176	70
Anthocyanin22	anthocyanin content	177	64
Chlorosis10	leaf chlorosis	177	28
Chlorosis16	leaf chlorosis	176	84
Chlorosis22	leaf chlorosis	176	110
Emco5	protist disease resistance	86	69
Emoy	protist disease resistance	76	41
Emwa1	protist disease resistance	85	53
Hiks1	protist disease resistance	84	51
LES	leaf necrosis	95	21
LY	leaf necrosis	95	29
Leafroll10	leaf margin serrated	177	99
Leafroll16	rolled leaf	176	37
Leafroll22	rolled leaf	176	31
Noco2	protist disease resistance	87	48
avrB	bacterial disease resistance	87	55
avrPphB	bacterial disease resistance	90	46
avrRpm1	bacterial disease resistance	84	56
avrRpt2	bacterial disease resistance	89	72

analysis, we consider $k \in \{1, ..., 5\}$, and report the results for the covariate that shows the best genomic inflation λ_{GC}, i.e. such that λ_{GC} is closest to 1 (see Fig. S6).

Importantly, we only look at the significant hits found with this covariate, not in the other 4 runs. We find that including covariates reduces population structure in 18 out of 20 phenotypes.

S3.3 Results

This section contains more details on the results found with the `SiNIMin`-based approaches, and all comparison partners. As for the *A. thaliana* phenotypes, all methods but `SKATO-interact` could be run. We count the number of significant hits for all applied methods and for each of the phenotypes, Tab. S3. With our approach `SiNIMin-WY`, we detect significant hits in 36 significant gene-interactions, spanning 10 different phenotypes. We compare the number of hits that were found with the baselines for the 36 gene-interactions that contained significant hits, and refer to those gene interactions as *novel*, that were not detected by any of the baselines. This means that neither of the adjacent genes, nor the complete edge, was significant or contains a significant sub-part (such as an individually significant SNP or a significant segment). Lastly, we look into the false-negatives, i.e. the number of interactions that were obtained with `FastLMM-interact` method. This constitutes the only comparison partner that tests the same hypothesis, and that could be run on the *A. thaliana* data sets (Tab. S4). We observe that `SiNIMin-WY` misses only five out of 25 hits detected with `FastLMM-interact`. However, three out of those five hits come from a phenotype that shows very high inflation with `FastLMM-interact`, i.e. the three hits that `SiNIMin-WY` missed might correspond to spurious associations in `FastLMM-interact`, rather than true positive associations.

Detailed results on the individual interactions for every phenotype can be found in the supplementary

	genes	edges	SNP-i.	segment-i.
**A.thal.**	4,431	9,380	280,152	6,617,466

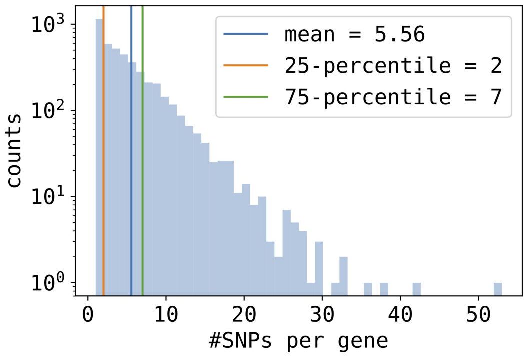

Figure S5: Dimensionality of the *A.thaliana* data. *(left)* The dimensions of the Interactome network that can be represented with SNP data of the data sets. The number of genes and edges correspond to those genes, that can be represented with SNPs, and edges between those genes. We furthermore count the number of all SNP-interactions and segment interactions within those edges, as described in the main text. *(right)* The distribution of gene-lengths, i.e. the number of SNPs mapped to a gene.

data files athal_edgeEpiWY_results.xlsx and athal_siniminWY_results.xlsx.

	best covar.	best λ_{gc}	base λ_{gc}
**Anthocyanin10**	5	1.167	1.457
**Anthocyanin16**	5	0.995	1.224
**Anthocyanin22**	5	1.098	1.430
**Chlorosis10**	1	1.032	1.032
**Chlorosis16**	5	0.991	0.988
**Chlorosis22**	5	1.101	1.766
**Emco5**	4	1.011	1.103
**Emoy**	3	1.106	1.166
**Emwa1**	3	1.062	1.350
**Hiks1**	2	1.155	1.229
**LES**	4	1.298	1.871
**LY**	3	1.275	2.351
**Leafroll10**	5	1.131	1.567
**Leafroll16**	5	1.152	1.449
**Leafroll22**	1	1.139	1.139
**Noco2**	4	1.128	1.336
**avrB**	2	1.101	1.586
**avrPphB**	5	1.244	1.762
**avrRpm1**	2	1.091	1.507
**avrRpt2**	2	1.252	1.327

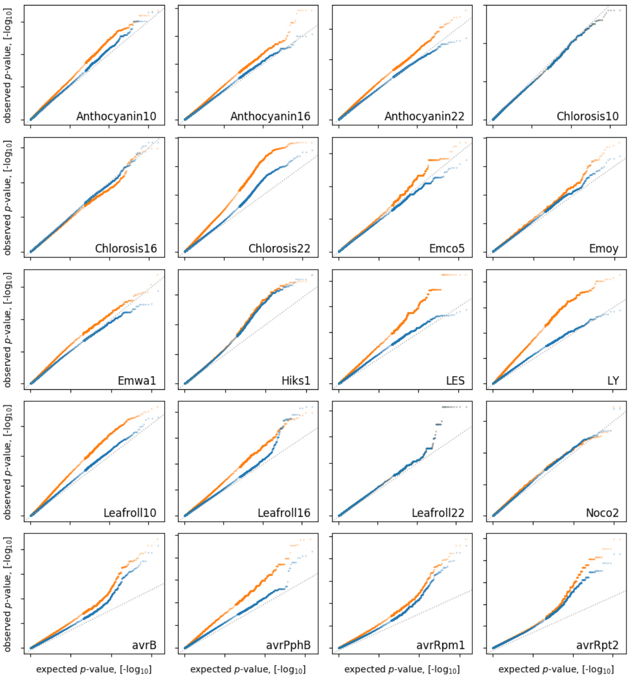

Figure S6: Analysis of inflation for the *A.thaliana* phenotypes using the `SiNIMin-WY` approach. The *(left)* table contains the baseline inflation factor if no covariates are used (column 'base λ_{gc}'), as well as the number of covariates that result in the best inflation (columns 'best covar.' and 'best λ_{gc}'). The figure on the *(right)* shows the corresponding Q-Q plots for the different phenotypes for the `SiNIMin-WY` approach, when using the best number of covariates (blue) versus no covariates (orange).

Table S3: The number of significant hits found with each of the methods on all *A. thaliana* phenotypes. The numbers in brackets correspond to the number of either genes or gene-interactions the significant hits map to.

	SiNIMin-WY	SiNIMin	edgeEpi-WY	edgeEpi	FASTCMH	FastLMM-interact	FastLMM-interval	FastLMM-edge	FastLMM-gene	FastLMM-singleSNP	SKATO-interact	SKATO-interval	SKATO-edge	SKATO-gene
**Anthocyanin10**	0 (0)	0 (0)	0 (0)	0 (0)	0 (0)	0 (0)	0 (0)	0 (0)	0 (0)	0 (0)	x	0 (0)	0 (0)	0 (0)
**Anthocyanin16**	0 (0)	0 (0)	0 (0)	0 (0)	0 (0)	0 (0)	0 (0)	0 (0)	0 (0)	0 (0)	x	0 (0)	0 (0)	0 (0)
**Anthocyanin22**	0 (0)	0 (0)	0 (0)	0 (0)	0 (0)	0 (0)	0 (0)	0 (0)	0 (0)	0 (0)	x	0 (0)	0 (0)	0 (0)
**Chlorosis10**	0 (0)	0 (0)	0 (0)	0 (0)	0 (0)	0 (0)	0 (0)	0 (0)	0 (0)	0 (0)	x	0 (0)	0 (0)	0 (0)
**Chlorosis16**	0 (0)	0 (0)	0 (0)	0 (0)	0 (0)	0 (0)	0 (0)	0 (0)	0 (0)	0 (0)	x	0 (0)	0 (0)	0 (0)
**Chlorosis22**	3 (2)	0 (0)	0 (0)	0 (0)	2 (1)	0 (0)	0 (0)	1 (1)	1 (1)	4 (2)	x	1 (1)	1 (1)	0 (0)
**Emco5**	0 (0)	0 (0)	0 (0)	0 (0)	0 (0)	0 (0)	0 (0)	0 (0)	0 (0)	0 (0)	x	15 (15)	15 (15)	2 (2)
**Emoy**	3 (2)	0 (0)	4 (2)	1 (1)	0 (0)	0 (0)	0 (0)	0 (0)	1 (1)	1 (1)	x	0 (0)	0 (0)	0 (0)
**Emwa1**	0 (0)	0 (0)	0 (0)	0 (0)	0 (0)	0 (0)	0 (0)	0 (0)	0 (0)	0 (0)	x	0 (0)	0 (0)	0 (0)
**Hiks1**	26 (7)	0 (0)	3 (3)	0 (0)	0 (0)	0 (0)	0 (0)	0 (0)	0 (0)	1 (1)	x	0 (0)	0 (0)	0 (0)
**LES**	0 (0)	0 (0)	0 (0)	0 (0)	0 (0)	0 (0)	0 (0)	0 (0)	1 (1)	0 (0)	x	0 (0)	0 (0)	0 (0)
**LY**	0 (0)	0 (0)	0 (0)	0 (0)	0 (0)	0 (0)	0 (0)	0 (0)	1 (1)	0 (0)	x	5 (5)	5 (5)	1 (1)
**Leafroll10**	0 (0)	0 (0)	1 (1)	0 (0)	0 (0)	0 (0)	0 (0)	0 (0)	0 (0)	0 (0)	x	0 (0)	0 (0)	0 (0)
**Leafroll16**	23 (1)	10 (1)	0 (0)	0 (0)	0 (0)	0 (0)	0 (0)	0 (0)	0 (0)	1 (1)	x	0 (0)	0 (0)	0 (0)
**Leafroll22**	16 (1)	16 (1)	0 (0)	0 (0)	0 (0)	0 (0)	0 (0)	0 (0)	0 (0)	0 (0)	x	0 (0)	0 (0)	0 (0)
**Noco2**	2 (1)	0 (0)	0 (0)	0 (0)	0 (0)	0 (0)	0 (0)	0 (0)	0 (0)	0 (0)	x	0 (0)	0 (0)	0 (0)
**avrB**	70 (6)	29 (4)	35 (5)	27 (5)	9 (8)	341 (6)	24 (2)	2 (2)	1 (1)	4 (2)	x	0 (0)	0 (0)	1 (1)
**avrPphB**	15 (1)	10 (1)	4 (1)	4 (1)	1 (1)	13 (1)	6 (1)	1 (1)	1 (1)	2 (1)	x	0 (0)	0 (0)	1 (1)
**avrRpm1**	74 (6)	43 (5)	37 (5)	28 (5)	9 (8)	827 (6)	20 (2)	2 (2)	2 (2)	4 (2)	x	2 (2)	2 (2)	1 (1)
**avrRpt2**	114 (9)	82 (9)	108 (11)	102 (11)	1 (1)	50310 (12)	57 (2)	10 (10)	2 (2)	6 (2)	x	7 (7)	7 (7)	3 (3)

Table S4: Comparison of `SiNIMin-WY` and its closest comparison partner, `FastLMM-interact`. Columns III to V contain the number of hits found with `SiNIMin-WY` but not `FastLMM-interact` (III), the hits found with both methods (IV) and the hits found with `FLMM-interact` but not with `SiNIMin-WY` (V). It should be noted that for avrRpt2 the inflation is very high with `FastLMM-interact`, while `SiNIMin-WY` results in a better control of inflation. Hence it might be possible that the three hits missed with `SiNIMin-WY` are due to the inflation.

	SiNIMin-WY λ_{GC}	SiNIMin-WY / FastLMM-interact	SiNIMin-WY ∩ FastLMM-interact	FastLMM-interact / SiNIMin-WY	FastLMM-interact λ_{GC}
**Anthocyanin10**	1.167	0	0	0	1.139
**Anthocyanin16**	0.995	0	0	0	1.172
**Anthocyanin22**	1.098	0	0	0	1.046
**Chlorosis10**	1.032	0	0	0	1.100
**Chlorosis16**	0.991	0	0	0	0.903
**Chlorosis22**	1.101	2	0	0	1.130
**Emco5**	1.011	0	0	0	1.091
**Emoy**	1.106	2	0	0	1.431
**Emwa1**	1.062	0	0	0	1.365
**Hiks1**	1.155	7	0	0	1.251
**LES**	1.298	0	0	0	1.030
**LY**	1.275	0	0	0	1.049
**Leafroll10**	1.131	0	0	0	1.076
**Leafroll16**	1.152	1	0	0	1.135
**Leafroll22**	1.139	1	0	0	1.244
**Noco2**	1.128	1	0	0	1.074
**avrB**	1.101	1	5	1	1.071
**avrPphB**	1.244	0	1	0	1.116
**avrRpm1**	1.091	1	5	1	1.041
**avrRpt2**	1.252	0	9	3	1.447

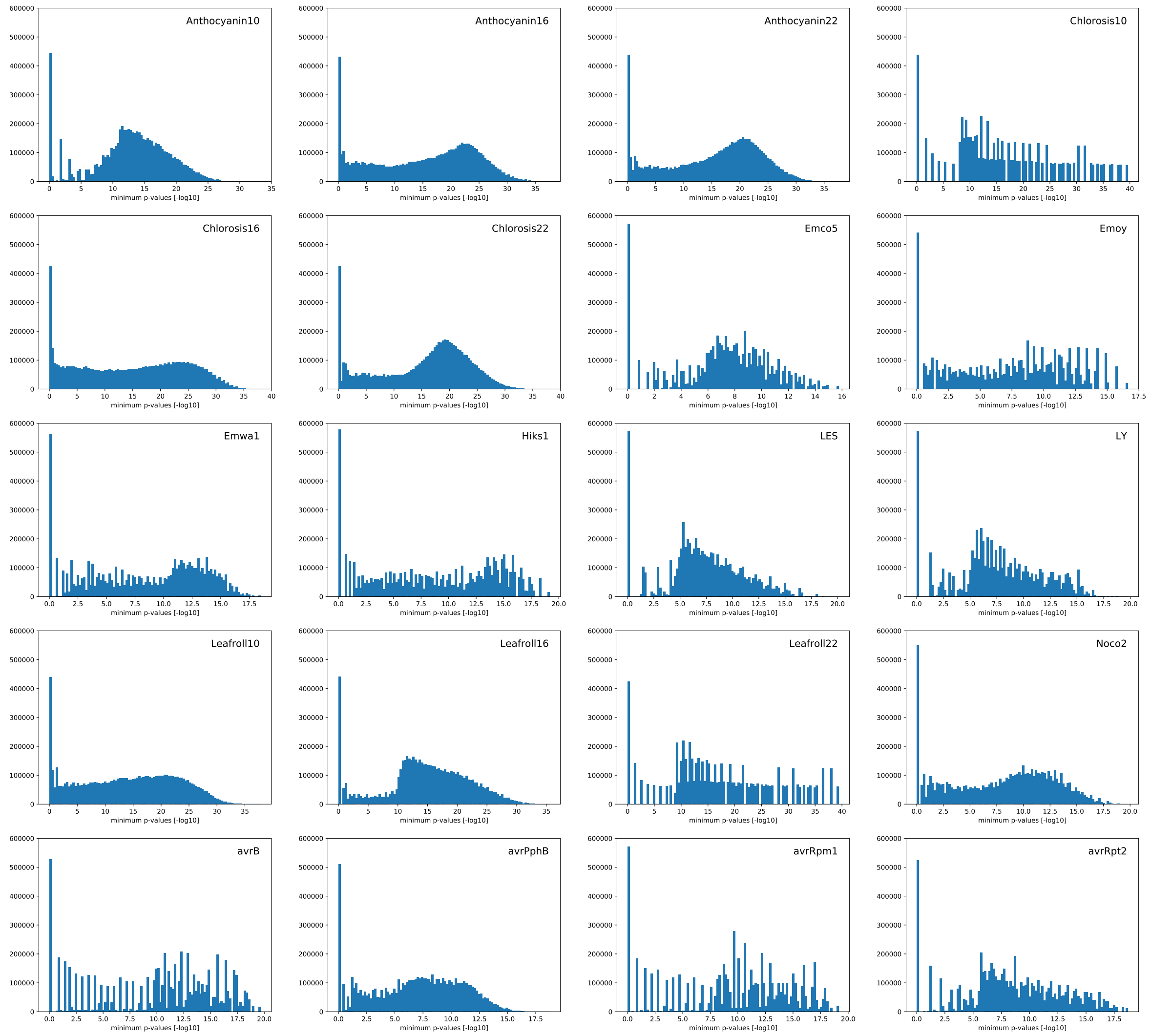

Figure S7: Distributions of all minimum p-values for all 6’617’466 possible segment pairs. We observe that the distributions exhibit a bimodal behavior, with one of the modes around p-values of 1.0. Patterns with high minimum p-values are likely to be pruned with our approaches. Note that this comprises *all* segment interactions. In our proposed approaches `SiNIMin` and `SiNIMin-WY` we conduct a *closed* mining of the segment interactions, that means that not necessarily all of the above patterns are tested.

Table S5: The number of testable sets found with the `SiNIMin` approach for each of the *A. thaliana* phenotypes. In total, there exist 6,617,466 possible patterns. We observe that our proposed method results in a reduction of the correction factor of at least 50% across all phenotypes, compared to the standard Bonferroni correction.

	`SiNIMin`: number of testable patterns
**Anthocyanin10**	3,062,434
**Anthocyanin16**	2,843,945
**Anthocyanin22**	2,941,953
**Chlorosis10**	3,185,203
**Chlorosis16**	2,725,857
**Chlorosis22**	2,961,050
**Emco5**	1,401,385
**Emoy**	1,513,745
**Emwa1**	1,663,305
**Hiks1**	1,654,315
**LES**	3,185,203
**LY**	1,346,981
**Leafroll10**	2,769,083
**Leafroll16**	3,125,226
**Leafroll22**	3,179,362
**Noco2**	1,675,602
**avrB**	1,929,393
**avrPphB**	1,425,788
**avrRpm1**	1,939,403
**avrRpt2**	1,562,643

Table S6: List of all intervals detected with `SiNIMin-WY` within the gene-gene interactions for the novel *A. thaliana* hits. The '-' symbol separates segments from the first gene and segments from the second gene. SNPs in a segment are represented as SNP_1:SNP_2:...:SNP_N. The coloring indicates the position of the SNP within the gene, where green: coding sequence, purple: 5'-UTR and orange: 3'-UTR. For some of the genes, there exist multiple splice variants. In those cases, the following color-scheme is applied to indicate that a SNP lies in different positions for different variants of the gene: pink: coding sequence, 3'-UTR, blue: coding sequence, transposable element, and brown: coding sequence, 5'-UTR. We find that roughly 46% of SNPs in the segments lie in intronic regions, 46% lie in the coding sequence, 2% lie in the 3'-UTR, 5% in the 5'UTR, and 1% fall within transposable elements.

dataset	gene-gene interaction	intervals
Leafroll22	AT3G18490 - AT5G42980	Chr3_6349128:Chr3_6349168 - Chr5_17242768:Chr5_17242977:Chr5_17243009:Chr5_17243136:Chr5_17243295:Chr5_17243309:Chr5_17243531
		Chr3_6349128:Chr3_6349168 - Chr5_17242977:Chr5_17243009:Chr5_17243136:Chr5_17243295:Chr5_17243309:Chr5_17243531
		Chr3_6349128:Chr3_6349168 - Chr5_17243009:Chr5_17243136:Chr5_17243295:Chr5_17243309:Chr5_17243531
		Chr3_6349128:Chr3_6349168 - Chr5_17243136:Chr5_17243295:Chr5_17243309:Chr5_17243531
		Chr3_6349128:Chr3_6349168:Chr3_6350713 - Chr5_17242768:Chr5_17242977:Chr5_17243009:Chr5_17243136:Chr5_17243295:Chr5_17243309:Chr5_17243531
		Chr3_6349128:Chr3_6349168:Chr3_6350713 - Chr5_17242977:Chr5_17243009:Chr5_17243136:Chr5_17243295:Chr5_17243309:Chr5_17243531
		Chr3_6349128:Chr3_6349168:Chr3_6350713 - Chr5_17243009:Chr5_17243136:Chr5_17243295:Chr5_17243309:Chr5_17243531
		Chr3_6349128:Chr3_6349168:Chr3_6350713 - Chr5_17243136:Chr5_17243295:Chr5_17243309:Chr5_17243531
		Chr3_6349168 - Chr5_17242768:Chr5_17242977:Chr5_17243009:Chr5_17243136:Chr5_17243295:Chr5_17243309:Chr5_17243531
		Chr3_6349168 - Chr5_17242977:Chr5_17243009:Chr5_17243136:Chr5_17243295:Chr5_17243309:Chr5_17243531
		Chr3_6349168 - Chr5_17243009:Chr5_17243136:Chr5_17243295:Chr5_17243309:Chr5_17243531
		Chr3_6349168 - Chr5_17243136:Chr5_17243295:Chr5_17243309:Chr5_17243531
		Chr3_6349168:Chr3_6350713 - Chr5_17242768:Chr5_17242977:Chr5_17243009:Chr5_17243136:Chr5_17243295:Chr5_17243309:Chr5_17243531
		Chr3_6349168:Chr3_6350713 - Chr5_17242977:Chr5_17243009:Chr5_17243136:Chr5_17243295:Chr5_17243309:Chr5_17243531
		Chr3_6349168:Chr3_6350713 - Chr5_17243009:Chr5_17243136:Chr5_17243295:Chr5_17243309:Chr5_17243531
		Chr3_6349168:Chr3_6350713 - Chr5_17243136:Chr5_17243295:Chr5_17243309:Chr5_17243531
Noco2	AT2G01950 - AT3G43850	Chr2_442125 - Chr3_15703678:Chr3_15703736:Chr3_15703862
		Chr2_442125 - Chr3_15703736:Chr3_15703862
Chlorosis22	AT1G74490 - AT2G41100	Chr1_27995475 - Chr2_17137509:Chr2_17137635:Chr2_17137941
		Chr1_27995475 - Chr2_17137635:Chr2_17137941
Hiks1	AT1G15760 - AT5G43460	Chr1_5425743 - Chr5_17460589:Chr5_17460643:Chr5_17460673
		Chr1_5425743 - Chr5_17460564:Chr5_17460589:Chr5_17460643:Chr5_17460673
		Chr1_5425783 - Chr5_17460589:Chr5_17460643:Chr5_17460673
		Chr1_5425783 - Chr5_17460589:Chr5_17460643
		Chr1_5425743:Chr1_5425783 - Chr5_17460589:Chr5_17460643:Chr5_17460673
		Chr1_5425743:Chr1_5425783 - Chr5_17460589:Chr5_17460643
		Chr1_5425743:Chr1_5425783 - Chr5_17460564:Chr5_17460589:Chr5_17460643:Chr5_17460673
		Chr1_5425743:Chr1_5425783 - Chr5_17460564:Chr5_17460589:Chr5_17460643
		Chr1_5425783 - Chr5_17460564:Chr5_17460589:Chr5_17460643:Chr5_17460673
		Chr1_5425783 - Chr5_17460564:Chr5_17460589:Chr5_17460643
Hiks1	AT4G16845 - AT5G57380	Chr4_9476180:Chr4_9476218 - Chr5_23247572:Chr5_23248305:Chr5_23248386
Hiks1	AT4G19030 - AT5G43460	Chr4_10422057 - Chr5_17460589:Chr5_17460643:Chr5_17460673
		Chr4_10422057 - Chr5_17460564:Chr5_17460589:Chr5_17460643:Chr5_17460673
avrRpm1	AT1G15750 - AT1G17380	Chr1_5419701 - Chr1_5955434:Chr1_5956670:Chr1_5957009
		Chr1_5419701 - Chr1_5955158:Chr1_5955434:Chr1_5956670:Chr1_5957009
Leafroll16	AT5G25150 - AT5G45600	Chr5_8677973:Chr5_8678024:Chr5_8679390:Chr5_8679813:Chr5_8679845 - Chr5_18489086:Chr5_18489244
		Chr5_8677973:Chr5_8678024:Chr5_8679390:Chr5_8679813:Chr5_8679845 - Chr5_18489086:Chr5_18489244:Chr5_18489572
		Chr5_8677973:Chr5_8678024:Chr5_8679390:Chr5_8679813:Chr5_8679845:Chr5_8679904:Chr5_8681591 - Chr5_18489086:Chr5_18489244
		Chr5_8677973:Chr5_8678024:Chr5_8679390:Chr5_8679813:Chr5_8679845:Chr5_8679904:Chr5_8681591 - Chr5_18489086:Chr5_18489244:Chr5_18489572
		Chr5_8679813:Chr5_8679845:Chr5_8679904:Chr5_8681591 - Chr5_18489086:Chr5_18489244:Chr5_18489572
		Chr5_8679813:Chr5_8679845:Chr5_8679904:Chr5_8681591 - Chr5_18489086:Chr5_18489244
		Chr5_8678024:Chr5_8679390:Chr5_8679813:Chr5_8679845:Chr5_8679904:Chr5_8681591 - Chr5_18489086:Chr5_18489244
		Chr5_8678024:Chr5_8679390:Chr5_8679813:Chr5_8679845:Chr5_8679904:Chr5_8681591 - Chr5_18489086:Chr5_18489244:Chr5_18489572
		Chr5_8679390:Chr5_8679813:Chr5_8679845:Chr5_8679904:Chr5_8681591 - Chr5_18489086:Chr5_18489244
		Chr5_8679390:Chr5_8679813:Chr5_8679845:Chr5_8679904:Chr5_8681591 - Chr5_18489086:Chr5_18489244:Chr5_18489572
		Chr5_8679845:Chr5_8679904:Chr5_8681591 - Chr5_18488982:Chr5_18489086:Chr5_18489244:Chr5_18489572
		Chr5_8679845:Chr5_8679904:Chr5_8681591 - Chr5_18488982:Chr5_18489086:Chr5_18489244
		Chr5_8679845:Chr5_8679904:Chr5_8681591 - Chr5_18489086:Chr5_18489244:Chr5_18489572
		Chr5_8679845:Chr5_8679904:Chr5_8681591 - Chr5_18489086:Chr5_18489244
		Chr5_8679813:Chr5_8679845:Chr5_8679904:Chr5_8681591 - Chr5_18488982:Chr5_18489086:Chr5_18489244:Chr5_18489572
		Chr5_8679390:Chr5_8679813:Chr5_8679845:Chr5_8679904:Chr5_8681591 - Chr5_18488982:Chr5_18489086:Chr5_18489244
		Chr5_8679390:Chr5_8679813:Chr5_8679845:Chr5_8679904:Chr5_8681591 - Chr5_18488982:Chr5_18489086:Chr5_18489244:Chr5_18489572
		Chr5_8678024:Chr5_8679390:Chr5_8679813:Chr5_8679845:Chr5_8679904:Chr5_8681591 - Chr5_18488982:Chr5_18489086:Chr5_18489244:Chr5_18489572
		Chr5_8678024:Chr5_8679390:Chr5_8679813:Chr5_8679845:Chr5_8679904:Chr5_8681591 - Chr5_18488982:Chr5_18489086:Chr5_18489244
		Chr5_8677973:Chr5_8678024:Chr5_8679390:Chr5_8679813:Chr5_8679845:Chr5_8679904:Chr5_8681591 - Chr5_18488982:Chr5_18489086:Chr5_18489244:Chr5_18489572
		Chr5_8677973:Chr5_8678024:Chr5_8679390:Chr5_8679813:Chr5_8679845:Chr5_8679904:Chr5_8681591 - Chr5_18488982:Chr5_18489086:Chr5_18489244
		Chr5_8679813:Chr5_8679845:Chr5_8679904:Chr5_8681591 - Chr5_18488982:Chr5_18489086:Chr5_18489244
		Chr5_8677973:Chr5_8678024:Chr5_8679390:Chr5_8679813 - Chr5_18489086:Chr5_18489244:Chr5_18489572
avrB	AT1G15750 - AT1G17380	Chr1_5419701 - Chr1_5955158:Chr1_5955434:Chr1_5956670:Chr1_5957009
		Chr1_5419701 - Chr1_5955434:Chr1_5956670:Chr1_5957009

S4 Study of migraine subtypes

S4.1 Data preprocessing

As described in the main text, we pool data from five different migraine cohorts: Dutch cohort with aura (DMA), Dutch cohort without aura (DMO), German cohort with aura (GMA), German cohort without aura (GMA) and Finnish cohort with aura (FMA). We combine all five cohorts, and assign each sample affected by migraine with aura the label 1, and each sample suffering from migraine without aura the label 0, thereby creating a binary pehnotype vector. This gives rise to the *MaMo* cohort. We repeat this procedure, this time combining migraine patients with the same nationality, resulting in one data set of Dutch patients (*dMaMo*) and one of German patients (*gMaMo*). We use the InBio network [10] to derive interactions, and map SNPs to genes that fall within a 50kb window up- and downstream of the genes (see Tab. S7). The number of SNPs mapped to a gene varies for each data set, with a median of 2 SNPs per gene (see Fig. S8)

Table S7: Dimensions of migraine cohorts. The column 'snps' indicates the number of SNPs with minor allele frequency less than 0.05 that can be mapped to a gene (including a 50kb window up- and downstream of the gene). The column 'genes' indicates the number of genes that can be encoded in this way, and 'edges' corresponds to the number of interactions between the encoded genes in the InBio network, i.e. the number of interactions among which segment interactions will be tested. The columns 'snp interactions' and 'segment interactions' contain the number of interactions between SNPs and segments, that will be tested with the `edgeEpi`-based and `SiNIMin`-based approaches.

dataset	samples	cases	snps	genes	edges	SNP interactions	segment interactions
**MaMo**	5,013	2,275	15,935	7,994	81,793	629,191	8,743,261
**dMaMo**	1,849	734	16,285	8,092	82,567	646,407	9,133,634
**gMaMo**	2,231	1,071	15,947	7,993	81,057	629,923	8,502,931

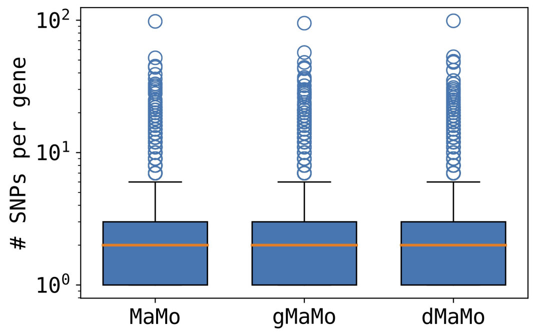

Figure S8: The number of SNPs that are mapped to the genes for each of the data sets. The median number of SNPs per gene is 2 for all three data sets.

S4.2 Control of genomic inflation

We generate global covariates for each sample using the MaMo data set in a two-step process: First, we do a principal component analysis (PCA) on the variance-standardized relationship matrix using *all* SNPs in the data set, not just those with minor allele frequency below 5%. Second, since our methods require discrete covariate classes, we cluster the principal components with *k-means* clustering.

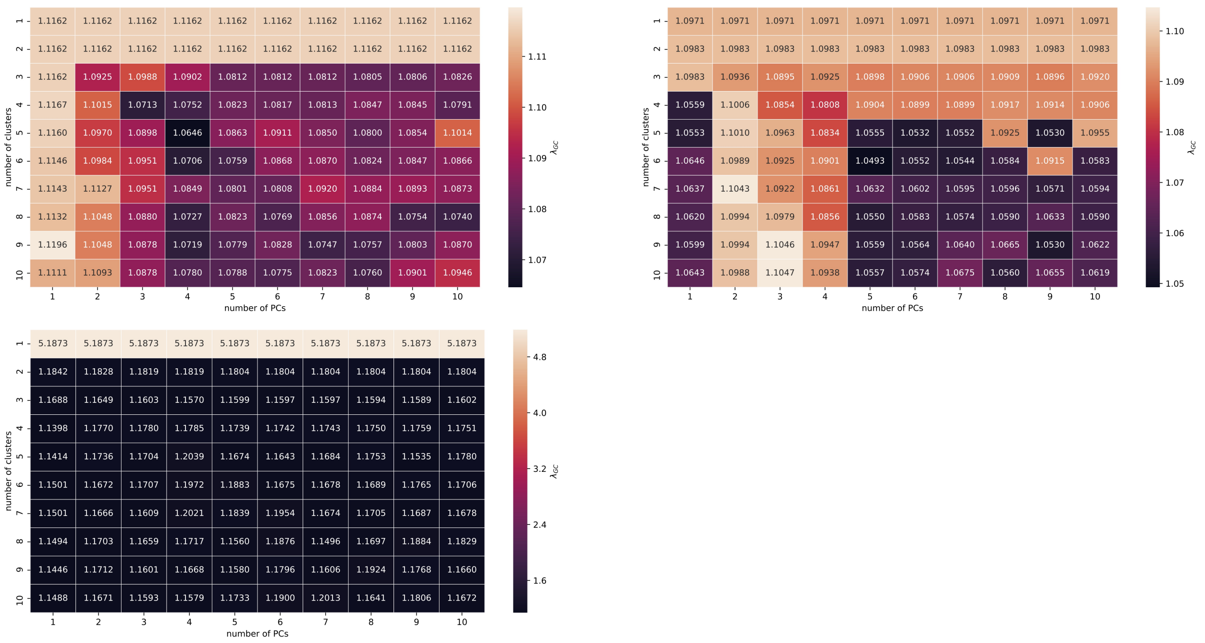

Figure S9: Search for hyperparamters in `EdgeEpiWY`. The genomic inflation factors for the `edgeEpi-WY` analysis of the dMaMo (top-left), gMaMo (top-right) and MaMo (bottom-left) when varying the number of principal components and the number of clusters to derive the categorical covariates.

Hence the number and composition of the resulting covariates depends on two parameters: (i) the number of principal components q, and (ii) the value of k in the clustering. We do a grid-search of the hyperparameter-space, where $q, k \in \{1, ..., 10\}$. For each run, we evaluate the genomic inflation factor λ_{GC}, and chose the one with λ_{GC} closest to 1 as our final result. Importantly, we only look at the significant hits found in this data set, not in the other 99 runs. The results of the hyperparameter grid search are illustrated in Fig. S9 for the `EdgeEpi-WY` analysis, in Fig S10 for the `SiNIMin-WY` analysis. We use the setting with the λ_{GC} value closest to 1 for each of the three cohorts, and generate the corresponding Q-Q plots (Fig. S11). Especially for the MaMo cohort, that contains samples from three different nationalities (dutch, finish, german), we observe severe inflation ($\lambda_{GC} = 5.19$ for `edgeWpi-WY`, $\lambda_{GC} = 4.85$ for `SiNIMin-WY`) if no correction for confounding is applied. Including covariates results in a reduction of the inflation. For dMaMo and gMaMo, we observe less severe inflation. However, including covariates diminishes the inflation, as measured by the genomic inflation factor λ_{GC}.

S4.3 Results

Here we give additional details to the results found on the different migraine cohorts with our proposed `SiNIMin`-based approaches, as well as the baseline comparison partners. As for the migraine cohorts, `SKATO-interact` and `FastLMM-interact` could not be executed within 120h, which is our upper time limit. The same is the case for the dMaMo data sets and `SKATO-segments` and `SKATO-edge`. In Tab. S8 we report the number of significant hits that were detected with each of the baseline methods, where 'x' indicates that a method could not be run. The counts indicate the number of significant hits, where the type of the significant entity depends on the method, e.g. for `SiNIMin` they corresponds to segment interactions, for `SKATO-edge` they correspond to edges, and so on. The number in brackets indicates the number of next-larger network entities this can be mapped to, i.e. nodes or edges.

Tab. S9 shows an analysis of false-negatives for network-based methods. Since we could not obtain results using baselines that test segment interactions, the closest comparison partners are the `FastLMM-edge`

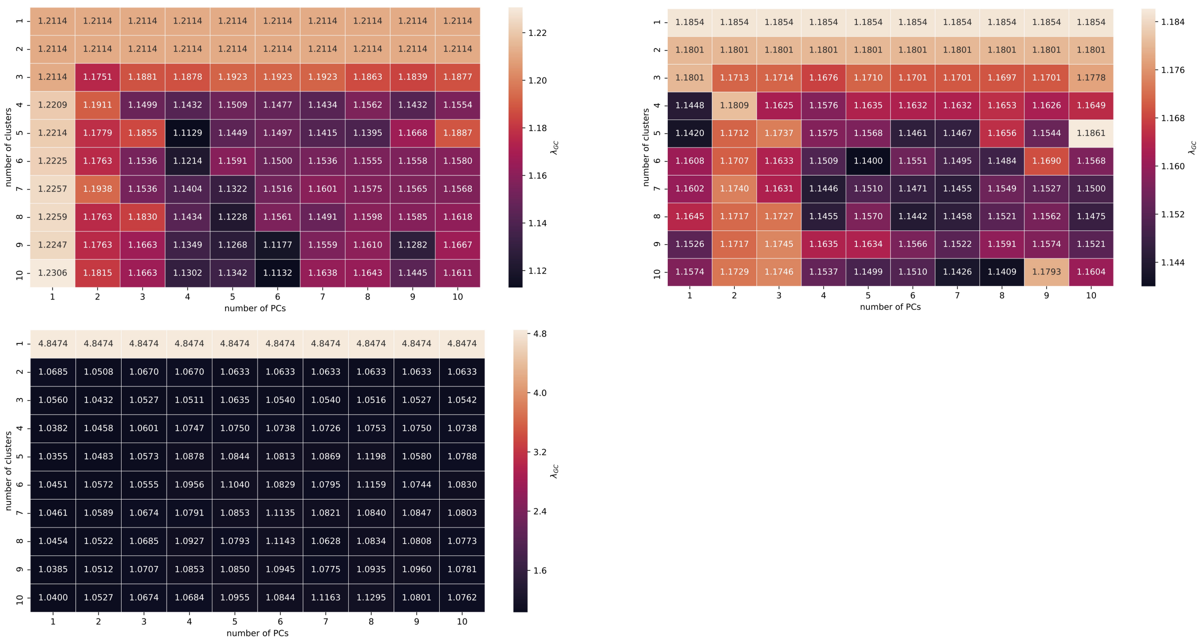

Figure S10: Search for hyperparamters in `SiNIMin-WY`. The genomic inflation factors for the `edgeEpi-WY` analysis of the dMaMo (top-left), gMaMo (top-right) and MaMo (bottom-left) when varying the number of principal components and the number of clusters to derive the categorical covariates.

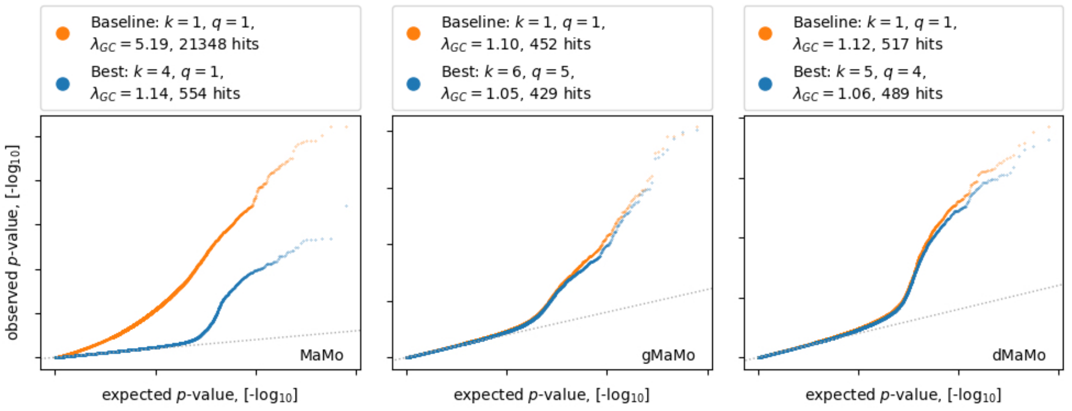

Figure S11: Q-Q plots for the `edgeEpi-WY` analysis. Each Q-Q plot contains the p-value distribution for one population. The orange dots constitute the baseline, that is `EdgeEpi-WY` p-values when no covariate is used, and hence no correction for population structure takes place. The blue dots represent `EdgeEpi-WY` p-values when the number of clusters k and principal components q is optimized to result in minimal in-/deflation. The legends indicate the number of clusters k in k-means clustering, the number of principal components q, the genomic inflation factor λ_{GC}, as well as the number of significant hits.

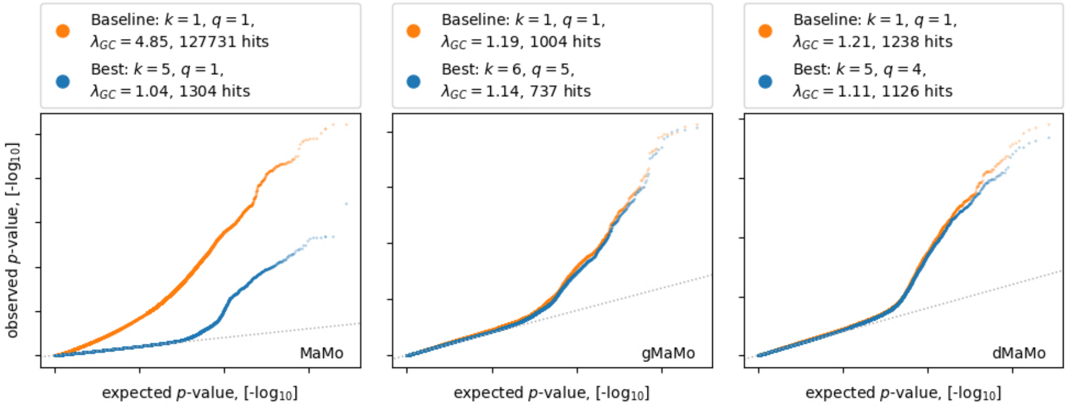

Figure S12: Q-Q plots for the `SiNIMin-WY` analysis. Each Q-Q plot contains the p-value distribution for one population. The orange dots constitute the baseline, that is `SiNIMin-WY` p-values when no covariate is used, and hence no correction for population structure takes place. The blue dots represent `SiNIMin-WY` p-values when the number of clusters k and principal components q is optimized to result in minimal in-/deflation. The legends indicate the number of clusters k in k-means clustering, the number of principal components q, the genomic inflation factor λ_{GC}, as well as the number of significant hits.

Table S8: Number of significant hits found with different methods. The first count contains the number of significant hits (e.g. segment interactions, segments, ...), the count in brackets corresponds to the number of gene-interactions they. For example, for MaMo, we found 33 significant SNP-segment interactions that map to 9 gene-interactions. Methods marked with 'x' did not finish within 120 hours.

	SiNIMin-WY	SiNIMin	edgeEpi-WY	edgeEpi	FASTCMH	FastLMM-interact	FastLMM-segment	FastLMM-edge	FastLMM-gene	FastLMM	SKATO-interact	SKATO-segment	SKATO-edge	SKATO-gene
**MaMo**	1304 (199)	1006 (166)	554 (209)	527 (202)	44 (44)	x	35 (22)	155 (155)	18 (18)	27 (27)	x	20 (20)	181 (181)	9 (9)
**gMaMo**	737 (149)	429 (93)	429 (216)	319 (169)	13 (13)	x	40 (12)	228 (228)	10 (10)	15 (15)	x	40 (40)	179 (179)	13 (13)
**dMaMo**	1126 (219)	819 (181)	489 (220)	467 (215)	26 (26)	x	40 (17)	203 (203)	16 (16)	21 (20)	x	x	x	19 (19)

and `SKATO-edge`. We consider a false negative for method A as those hits that were detected with method B, but not method A. In Tab. S9, the row contains the method for which false-negatives should be analysed. For example in the MaMo cohort, there are 33 hits that are detected with `SiNIMin-WY`, but not with `SiNIMin`. We observe that both `SiNIMin` and `SiNIMin-WY` miss many interactions compared to their SNP-based counterparts `edgeEpi` and `edgeEpi-WY`, indicating that for IHGC, interactions empirically seem to take place at the level of individual SNPs, not interactions between neighbouring SNPs under a model of genetic heterogeneity.

Detailed results on the individual interactions for every cohort can be found in the supplementary data files (ihgc_edgeEpiWY_results.xlsx, ihgc_siniminWY_results.xlsx).

S4.4 Analysis of gender covariates

In this analysis, we test different versions of covariates. Specifically, for each sample in the three IHGC cohorts we use gender as a covariate. We create three different types of covariates, namely: (i) PC only, (ii) gender only, and (iii) PC plus gender. The first one corresponds to the analysis in the main part of the paper, and in the subsections above. The second analysis creates a covariate vector with two different

Table S9: False negative analysis of the different methods that consider network information. Each of the three tables contain results for one of the three migraine data sets. The rows contain the method for which false-negatives are checked, the columns contain the comparison partners. For example, `SiNIMin-WY` detected 33 interactions that could not be detected with `SiNIMin`. Note that for dMaMo, the `SKATO-edge` did not run within 120 h, which is why there are no results listed for this method.

MaMo	SiNIMin-WY	SiNIMin	edgeEpi	edgeEpi-WY	FastLMM-edge	SKATO-edge
**SiNIMin-WY**	0	0	4	11	27	33
**SiNIMin**	33	0	36	43	28	48
**edgeEpi**	1	0	0	7	27	31
**edgeEpi-WY**	1	0	0	0	27	28
**FastLMM-edge**	71	39	74	81	0	47
**SKATO-edge**	51	33	52	56	21	0

gMaMo	SiNIMin-WY	SiNIMin	edgeEpi	edgeEpi-WY	FastLMM-edge	SKATO-edge
**SiNIMin-WY**	0	0	20	67	154	118
**SiNIMin**	56	0	76	123	174	131
**edgeEpi**	0	0	0	47	145	105
**edgeEpi-WY**	0	0	0	0	121	94
**FastLMM-edge**	75	39	86	109	0	42
**SKATO-edge**	88	45	95	131	91	0

dMaMo	SiNIMin-WY	SiNIMin	edgeEpi	edgeEpi-WY	FastLMM-edge	SKATO-edge
**SiNIMin-WY**	0	0	3	4	35	0
**SiNIMin**	38	0	34	39	55	0
**edgeEpi**	7	0	0	5	33	0
**edgeEpi-WY**	3	0	0	0	32	0
**FastLMM-edge**	51	33	45	49	0	0
**SKATO-edge**	219	181	215	220	203	0

classes, corresponding to the gender of each sample. The third is the combination of both. To generate a discretized version of the covariates, we append the binary gender-indicator to the principal components for each sample, and conduct a k-means clustering on the resulting matrix. We report the best genomic inflations for each data set and each covariate type in Table S11. We observe that including gender as a covariate does not improve the genomic inflation factor λ_{GC}.

Table S10: List of the novel segment interactions detected. As stated in the main text, only interactions between length-1 segments turned out to be novel (there exist significant segment-interactions between segments of lengths > 1, but due to the conservative criterion of how we evaluate novelty, at least one of the genes in each interaction could be found with a competitor method). The '-' symbol separates segments from the first and second gene in column 'gene-gene interaction', respectively. SNPs marked in green lie within the gene, as opposed to the 50kb window up- and downstream of the gene.

dataset	gene-gene interaction	segment interactions
**gMaMo**	BMP4 - BMPR1B	rs7141833 - rs1296143
**gMaMo**	HAO1 - VDAC3	rs6140453 - rs16891290
		rs6140453 - rs12676957
**dMaMo**	EPHA6 - TIAM1	rs2027815 - rs539330

Table S11: The genomic inflations λ_{GC} for different types of covariates and the three IHGC data sets, obtained with `SiNIMin-WY`.

(a) `SiNIMin-WY`

covariates	gMaMo	dMaMo	MaMo
**PC**	1.14002	1.11294	1.03546
**gender**	1.19246	1.21617	4.83981
**gender+PC**	1.1712	1.18571	1.04933

(b) `EdgeEpi-WY`

	gMaMo	dMaMo	MaMo
**PC**	1.04927	1.0646	1.13985
**gender**	1.10632	1.11383	5.16597
**gender+PC**	1.05958	1.0739	1.14703

References

[1] Felipe Llinares-López, Laetitia Papaxanthos, Dean Bodenham, Damian Roqueiro, COPDGene Investigators, and Karsten Borgwardt. Genome-wide genetic heterogeneity discovery with categorical covariates. *Bioinformatics*, 33(12):1820–1828, 2017.

[2] Christoph Lippert, Jennifer Listgarten, Ying Liu, Carl M Kadie, Robert I Davidson, and David Heckerman. Fast linear mixed models for genome-wide association studies. *Nature methods*, 8(10):833, 2011.

[3] Christoph Lippert, Jing Xiang, Danilo Horta, Christian Widmer, Carl Kadie, David Heckerman, and Jennifer Listgarten. Greater power and computational efficiency for kernel-based association testing of sets of genetic variants. *Bioinformatics*, 30(22):3206–3214, 2014.

[4] Seunggeun Lee, Michael C Wu, and Xihong Lin. Optimal tests for rare variant effects in sequencing association studies. *Biostatistics*, 13(4):762–775, 2012.

[5] Christopher C Chang, Carson C Chow, Laurent CAM Tellier, Shashaank Vattikuti, Shaun M Purcell, and James J Lee. Second-generation plink: rising to the challenge of larger and richer datasets. *Gigascience*, 4(1):s13742–015, 2015.

[6] Arabidopsis Interactome Mapping Consortium et al. Evidence for network evolution in an arabidopsis interactome map. *Science*, 333(6042):601–607, 2011.

[7] Vivek Krishnakumar, Matthew R Hanlon, Sergio Contrino, Erik S Ferlanti, Svetlana Karamycheva, Maria Kim, Benjamin D Rosen, Chia-Yi Cheng, Walter Moreira, Stephen A Mock, et al. Araport: the arabidopsis information portal. *Nucleic acids research*, 43(D1):D1003–D1009, 2014.

[8] Susanna Atwell, Yu S Huang, Bjarni J Vilhjálmsson, Glenda Willems, Matthew Horton, Yan Li, Dazhe Meng, Alexander Platt, Aaron M Tarone, Tina T Hu, et al. Genome-wide association study of 107 phenotypes in arabidopsis thaliana inbred lines. *Nature*, 465(7298):627, 2010.

[9] Ümit Seren, Dominik Grimm, Joffrey Fitz, Detlef Weigel, Magnus Nordborg, Karsten Borgwardt, and Arthur Korte. Arapheno: a public database for arabidopsis thaliana phenotypes. *Nucleic acids research*, page gkw986, 2016.

[10] Taibo Li, Rasmus Wernersson, Rasmus B Hansen, Heiko Horn, Johnathan Mercer, Greg Slodkowicz, Christopher T Workman, Olga Rigina, Kristoffer Rapacki, Hans H Stærfeldt, et al. A scored human protein–protein interaction network to catalyze genomic interpretation. *Nature methods*, 14(1):61, 2017.
